# Supplementary material for: How do smoking cessation medicines compare with respect to their neuropsychiatric safety? A protocol for a systematic review, network meta-analysis and cost-effectiveness analysis
Source: BMJ Open. 2017 Jun 17;7(6):e015414. doi: 10.1136/bmjopen-2016-015414 (PMC5734370; doi:10.1136/bmjopen-2016-015414)
Supplement: Supplementary data [file bmjopen-2016-015414supp001.pdf]

Appendix 1. Summary of pharmacologic interventions by dose and formulation.

**Treatment and formulation**	**Lower dose**	**Standard dose**	**Higher dose**
Bupropion			
Oral extended release tablets	<150 mg bd	150 mg bd	>150 mg bd
Varenicline			
Tablets	<1 mg bd	1 mg bd	>1 mg bd
Nicotine replacement therapy			
Patch (16 hrs)	<15 mg (5 mg/16 hours or 10 mg/16 hours)	15 mg (15 mg/16 hours)	>15 mg (25 mg/16 hours)
Patch (24 hrs)	< 14 mg (7 mg/24 hours)	14 mg (14 mg/24 hours)	>14 mg (21 mg/24 hours)
Gum		2 mg/piece (15 pieces daily)	4 mg/piece (15 pieces daily)
Nasal spray		0.5 mg/metered spray (up to 2 sprays/hour, 64 daily)	
Mouth spray		1 mg/metered spray (up to 4 sprays/hour, 64 daily)	
Lozenge	1 mg or 1.5 mg (1 lozenge/1-2 hours, 15 daily)	2 mg (1 lozenge/1-2 hours, 15 daily)	4 mg (1 lozenge/1-2 hours, 15 daily)
Sublingual tablet		2 mg per tablet (up to 1 tablet/hour, 40 daily)	2 mg per tablet (up to 2 tablets/hour, 40 daily)
Inhalator		10 mg/cartridge (12 cartridges daily)	15 mg/cartridge (6 cartridges daily)
Electronic Cigarette			
Electronic Inhaler		10mg/cartridge (5 cartridges daily)	15mg/cartridge (5 cartridges daily)

Note. Maximum doses in parentheses; mg = milligram; bd = twice daily. For nicotine replacement therapy the higher dose is generally used in smokers who smoke >20 cigarettes per day.

Appendix 2. Full electronic search strategy for observational studies of varenicline in Medline.

1 Smoking/

2 Tobacco/

3 Nicotine/

4 Tobacco Products/

5 Smoking/dt [Drug Therapy]

6 Smoking Cessation/

7 "Tobacco Use Cessation"/

8 "Tobacco Use Disorder"/

9 Smoking/pc [Prevention & Control]

10 (smoking or smoker*).ti,ab,kf.

11 (tobacco* or cigar* or cigarette* or nicotine).ti,ab,kf.

12 ((smoking or tobacco) adj5 (cessation or ceas* or quit* or stop* or giv* or prevent* or abstain* or abstin* or control*)).ti,ab,kf.

13 Varenicline/

14 Nicotinic Agonists/

15 varenicline.ti,ab,kf.

16 (champix or tabex or chantix).ti,ab,kf.

17 (nicotinic adj3 agonist*).ti,ab,kf.

18 (benzazepine* adj2 derivative*).ti,ab,kf.

19 nicotinic receptor partial agonist*.ti,ab,kf.

20 Epidemiologic Methods/ (30872)

21 epidemiologic studies/

22 case-control studies/ or retrospective studies/

23 cohort studies/ or follow-up studies/ or longitudinal studies/ or "national longitudinal study of adolescent health"/ or prospective studies/ or retrospective studies/

24 case-control studies/

25 Cross-Sectional Studies/

26 case control.ti,ab,kf.

27 (cohort adj (study or studies)).ti,ab,kf.

28 cohort analy*.ti,ab,kf.

29 (follow-up adj (study or studies)).ti,ab,kf.

30 (observational adj (study or studies)).ti,ab,kf.

31 (longitudinal or retrospective or prospective or cross sectional).ti,ab,kf.

32 (epidemiologic* adj (study or studies)).ti,ab,kf.

33 1 or 2 or 3 or 4 or 5 or 6 or 7 or 8 or 9 or 10 or 11 or 12

34 13 or 14 or 15 or 16 or 17 or 18 or 19

35 20 or 21 or 22 or 23 or 24 or 25 or 26 or 27 or 28 or 29 or 30 or 31 or 32

36 33 and 34 and 35
