# Supplementary material for: How do smoking cessation medicines compare with respect to their neuropsychiatric safety? A protocol for a systematic review, network meta-analysis and cost-effectiveness analysis
Source: BMJ Open. 2017 Jun 17;7(6):e015414. doi: 10.1136/bmjopen-2016-015414 (PMC5734370; doi:10.1136/bmjopen-2016-015414)
Supplement: Author's manuscript [file bmjopen-2016-015414.draft_revisions.pdf]

BMJ Open

# How do smoking cessation medicines compare with respect to their neuropsychiatric safety? A protocol for a systematic review, network meta-analysis and cost-effectiveness analysis

| | |
|---|---|
| Journal: | *BMJ Open* |
| Manuscript ID | bmjopen-2016-015414 |
| Article Type: | Protocol |
| Date Submitted by the Author: | 02-Dec-2016 |
| Complete List of Authors: | Thomas, Kyla; University of Bristol, School of Social and Community Medicine<br>Caldwell, Deborah; University of Bristol, School of Social and Community Medicine<br>Dalili, Michael; University of Bristol, School of Social and Community Medicine<br>Gunnell, DJ; University of Bristol, School of Social and Community Medicine<br>Munafò, Marcus; University of Bristol, MRC Integrative Epidemiology Unit; University of Bristol, UK Centre for Tobacco and Alcohol Studies, School of Experimental Psychology<br>Stevenson, Matt; University of Sheffield School of Health and Related Research<br>Welton, Nicky; University of Bristol, School of Social and Community Medicine |
| <b>Primary Subject Heading</b>: | Smoking and tobacco |
| Secondary Subject Heading: | Public health, Pharmacology and therapeutics |
| Keywords: | varenicline, bupropion, nicotine replacement therapy, electronic cigarettes, smoking cessation, network meta-analysis |
| | |

SCHOLARONE™
Manuscripts

*How do smoking cessation medicines compare with respect to their neuropsychiatric safety? A protocol for a systematic review, network meta-analysis and cost-effectiveness analysis*

Kyla H Thomas[1], Deborah Caldwell[1], Michael N Dalili[1], David Gunnell[1], Marcus R Munafò[2,3], Matt Stevenson[4], Nicky J Welton[1]

1 School of Social and Community Medicine, University of Bristol, Bristol, UK

2 MRC Integrative Epidemiology Unit, University of Bristol, Bristol, UK

3 UK Centre for Tobacco and Alcohol Studies, School of Experimental Psychology, University of Bristol, Bristol, UK

4 School of Health and Related Research, University of Sheffield, Sheffield, UK

Correspondence to
Dr Kyla H Thomas; kyla.thomas@bristol.ac.uk

# ABSTRACT

### Introduction

Cigarette smoking is one of the leading causes of early death in the UK and worldwide. Public health guidance recommends the use of varenicline, bupropion and nicotine replacement therapy (NRT) as smoking cessation aids in the UK. Additionally, the first electronic cigarette has been licensed for use as a smoking cessation medicine. However, there are ongoing concerns about the safety of these medicines. We present a protocol for a systematic review and network meta-analysis (NMA) to determine how these smoking cessation medicines compare to each other with respect to their neuropsychiatric safety in adult smokers. Secondary aims include updating the evidence regarding the effectiveness and cardiovascular safety of these medicines for use in a cost-effectiveness analysis.

### Methods and analysis

We will include randomised controlled trials and observational studies with control groups comparing monotherapy with varenicline, bupropion, NRT or electronic cigarette and combination therapies to each other, placebo or usual care. The primary composite safety outcome will be serious adverse events, defined as events that resulted in death, were life threatening, required hospitalisation, or resulted in significant disability or congenital/birth defect. The preferred effectiveness outcome will be sustained smoking cessation defined as abstinence for a minimum of six months as determined by biochemical validation. We will include trials identified by previous reviews and search relevant databases for newly published trials as well as contacting study authors to identify unpublished information. We will conduct fixed- and random-effects meta-analyses for each pairwise comparison of treatments and



For peer review only - http://bmjopen.bmj.com/site/about/guidelines.xhtml

For peer review only

outcome; where these estimates differ we will consider reasons for heterogeneity, quantified using the between-study variance ($\tau^2$). For each outcome, we will construct a NMA in a Bayesian framework which will be compared to the pair-wise results, allowing us to rank treatments. The effectiveness estimates from the NMA will be entered into a probabilistic economic model.

**Ethics and dissemination**

Ethics approval is not required for this evidence synthesis study as it involves analysis of secondary data from randomised controlled trials and observational studies. The review will make an important contribution to the knowledge base around the effectiveness, safety and cost-effectiveness of smoking cessation medicines. Results will be disseminated to the general public, healthcare practitioners and clinicians, academics, industry and policy makers.

**PROSPERO registration number** CRD42016041302

# STRENGTHS AND LIMITATIONS OF THIS STUDY

- This study will be the first comprehensive review of the neuropsychiatric safety of smoking cessation medicines in relation to each other and will include randomised controlled trials of any duration as well as observational studies with control groups. Including observational studies will allow us to identify sufficient data for the NMA and improve the precision of estimates of adverse events.

- This study will include trials of combined therapies of smoking cessation medicines, not included in previous network meta-analyses, as the analysis of both safety and efficacy data on co-prescribing could be of importance to regulators.

- This study will produce updated cost-effectiveness analyses to estimate which smoking cessation medicine is the most cost-effective in UK settings.

- We will use the Cochrane tool for assessing the risk of bias and consider using the risk of bias in non-randomized studies of interventions (ROBINS-I) tool to assess the risk of bias in randomised controlled trials and observational studies, respectively.

- This study will be limited to products licensed for use as smoking cessation aids in the UK.

# INTRODUCTION

Cigarette smoking is one of the leading causes of early death in the UK and worldwide [1,2]. Each year, more than 100,000 people will die in the UK from smoking related diseases [3]. NICE public health guidance (published in 2008) recommends the use of three medicines, varenicline, bupropion and nicotine



For peer review only - http://bmjopen.bmj.com/site/about/guidelines.xhtml

replacement therapy (NRT), as aids to quitting smoking in the UK [4]. Additionally, in late 2015 the Medicines and Healthcare Products Regulatory Agency (MHRA) approved the use of British American Tobacco's electronic cigarette "e-Voke" as a smoking cessation medicine [5]. Since the publication of the original NICE guidance, there have been ongoing concerns about the safety of the smoking cessation medicines, with particular respect to the neuropsychiatric safety of varenicline. Severe safety warnings (black triangle and black box warnings) regarding a potential increased risk of serious neuropsychiatric adverse events (depression, suicidal ideation and suicidal behaviour) in patients prescribed these medicines have previously been issued by regulatory agencies such as the MHRA in the UK and the Food and Drug Administration (FDA) in the US [6,7]. These safety warnings were based on spontaneous reports to the UK Yellow Card Scheme and the FDA Adverse Events Reporting Database. Previous research into the neuropsychiatric safety of these medicines has provided inconsistent findings, adding to the debate [8]. While the MHRA removed their black triangle warning in April 2016, the FDA's black box warnings remain on varenicline's labelling. With the MHRA's licensing of the first electronic cigarette as a smoking cessation medicine, and given the popularity of electronic cigarettes with an estimated 2.8 million adult users in Great Britain [9], it is important to review their effectiveness as a smoking cessation aid as well as their safety.

The health benefits of smoking cessation have been well documented. Varenicline has been shown to be the most clinically effective monotherapy for long-term smoking abstinence (>6 months) [10]. However, the number of prescription items of varenicline dispensed in England decreased by 25% from a peak of approximately one million prescriptions in 2011 to almost 742,000 prescriptions in 2013 [11], possibly reflecting ongoing fears among prescribers and patients regarding varenicline's neuropsychiatric safety. It is important for patients, prescribers and regulators to know how smoking cessation medicines compare with each other, with particular respect to their neuropsychiatric safety, to enable smokers wanting to quit and their healthcare professionals to make informed decisions about the risks and benefits of the different pharmacological treatments. To date, there has been no comprehensive analysis of the neuropsychiatric safety of the smoking cessation medicines; previous systematic reviews have focussed on comparisons of varenicline with placebo [12-14]. In addition, the cost-effectiveness of these medicines in UK settings has not been investigated using the most up to date evidence. These analyses are important to inform the overall risk-benefit evaluation of the different smoking cessation medicines.

The ongoing debate regarding the neuropsychiatric safety of drugs for smoking cessation among drug regulators, researchers, prescribers and patients may be due to the inconsistent research findings in this area [8]. Whereas studies without control groups (such as those using adverse event reporting data and case studies) [15-17] have reported increased risks of self-reported depression and suicidal or self-injurious behaviour in patients prescribed varenicline and bupropion, studies with control groups (such as observational cohort studies and experimental study designs, mainly randomised controlled trials [RCTs] and systematic reviews of



For peer review only - http://bmjopen.bmj.com/site/about/guidelines.xhtml

RCTs) have reported the opposite, and found no evidence of an increased risk of these severe neuropsychiatric outcomes in patients prescribed these medicines [12-14,18-21]. However, there are important limitations associated with each of these study designs. First, studies which use spontaneous adverse event reports are limited by several factors. These include the severity of the adverse event (severe adverse events are more likely to be reported than less serious events) [22], the length of time that the drug has been available (adverse events with newer drugs are more likely to be reported than events occurring with older drugs for the same indication) and media publicity about a drug (media reports often lead to increased reporting of adverse events to the Yellow Card Scheme, known as stimulated reporting) [23,24]. Second, observational cohort studies are prone to the effects of confounding by indication, which raises concerns about the validity of their findings [25]. Confounding by indication may occur if an observed association between smoking cessation medicines and serious psychiatric events such as suicide is explained if the patient case mix differs among those taking different smoking cessation medications [18,26,27]. In two of the earlier cohort studies, varenicline and bupropion were found to be associated with a decreased risk of death from all causes compared to NRT [18,19]; this reported protective effect was most likely due to residual confounding in the studies.

Experimental studies are less likely to suffer from uncontrolled confounding, however, to date, systematic reviews and meta-analyses of RCTs have mainly focussed on comparing the neuropsychiatric safety of varenicline monotherapy with placebo [12-14,28]. Although this is an important research question, patients are unlikely to be prescribed placebo in real life settings to help them quit smoking. Therefore the neuropsychiatric safety of varenicline compared to other smoking cessation drugs is likely to be of greater relevance to patients, prescribers and regulators. To date, there have been no comprehensive reviews of the neuropsychiatric safety of the smoking cessation medicines in relation to each other. In addition, there have been no recent cost-effectiveness analyses that have fully accounted for adverse events in order to determine which UK-licensed smoking cessation medicine is estimated to be the most cost-effective in UK settings. The aim of this study is to determine how smoking cessation medicines compare to each other with respect to their neuropsychiatric safety in adult smokers. Secondary aims include updating the evidence regarding the effectiveness and cardiovascular safety of these medicines for use in a cost-effectiveness analysis.

# METHODS AND ANALYSIS

## Criteria for screening studies

### Study designs

For the safety network meta-analyses, RCTs of any duration will be included in addition to observational studies with control groups. Uncontrolled observational studies (e.g., case reports and case series) will be excluded.



For peer review only - http://bmjopen.bmj.com/site/about/guidelines.xhtml

For peer review only

For the effectiveness network meta-analyses, we will include RCTs of greater than 6 months' duration in any setting, including, but not limited to, primary care practices, hospitals including inpatient and outpatient clinics, universities, workplace clinics, nursing or residential homes. Multi-arm trials will be included while crossover trials, non-randomised trials, quasi-randomised trials and interrupted time series analyses will be excluded from the effectiveness analyses.

## Participants

We will include smokers aged 18 years and over of all ethnicities who are seeking to quit smoking using UK-licensed smoking cessation therapies. This includes adult smokers accessing local authority stop smoking services. We will also include smokeless tobacco users. We will exclude studies with participants less than 18 years of age as varenicline, bupropion and electronic cigarettes are only licensed for use in adults in the UK. Non-smoking populations will be excluded as well as pregnant and breastfeeding women, as varenicline and bupropion are not licensed for use in these groups in the UK. If sufficient evidence is identified, we plan to consider the following subgroups in all analyses: those with psychiatric illness (for example depression, schizophrenia, bipolar disorder, substance misuse); cardiovascular disease (for example peripheral vascular disease, acute coronary syndromes and post myocardial infarction); Chronic Obstructive Pulmonary Disease; diabetics; heavy smokers (defined as people who smoke >20 cigarettes per day); and those with previous quit attempts.

## Interventions

The health technologies that we will assess include varenicline, bupropion, nicotine replacement therapy and electronic cigarette as monotherapy and in combination treatment (e.g. varenicline combined with NRT, varenicline combined with bupropion and bupropion compared to NRT). For NRT, combinations of different formulations given concurrently, for example patch and gum, will also be assessed. Different dosages of treatments will also be examined. Online supplementary appendix 1 summarises the main pharmacologic monotherapies for smoking cessation by formulation and dosage using the British National Formulary (BNF) September 2016 version (http://www.evidence.nhs.uk/formulary/bnf/current) and the MHRA public assessment report for the "e-Voke" [5]. Combination therapies and combinations of NRT formulations will also be included in the analyses.

We will exclude trials of alternative and complementary therapies (e.g., hypnotherapy, acupuncture, aromatherapy and herbal therapies), and psychotherapies (unless they are included as co-treatment with a pharmacologic intervention).

## Outcomes

### Safety analysis

The primary composite safety outcome will be serious adverse events, defined as events that resulted in death, were life threatening, required hospitalisation, or



For peer review only - http://bmjopen.bmj.com/site/about/guidelines.xhtml

resulted in significant disability or congenital/birth defect. Safety outcomes will be grouped under the following headings:

- Neuropsychiatric outcomes. The primary neuropsychiatric outcomes will be major adverse neuropsychiatric events (MANE) which will include: completed suicide; attempted suicide; suicidal ideation; depression; and seizures. Secondary neuropsychiatric outcomes will include: abnormal dreams; aggression; anxiety; fatigue; insomnia; irritability; sleep disorders; and somnolence.
- Cardiovascular outcomes. The primary cardiovascular outcomes will be major adverse cardiovascular events (MACE) which will include: cardiovascular death; nonfatal myocardial infarction (i.e. unstable angina); and non-fatal stroke based on the FDA definition [29]. Secondary cardiovascular outcomes will include: transient ischemic attack; congestive heart failure; palpitations; arrhythmias; and thromboembolism (deep vein thrombosis and pulmonary embolism).

Other outcomes will include adverse events such as nausea, headache, dry mouth, skin rash and pruritus.

### Effectiveness analysis

The primary effectiveness outcome will be sustained smoking cessation, defined as abstinence for a minimum of six months as determined by biochemically validated continuous or prolonged abstinence at the longest reported time point in intention to treat analyses, as this is the strictest definition of abstinence. Where these data are not available, we will accept point prevalence abstinence and self-report quit data. We will treat participants who drop out or are lost to follow-up as continuing smokers. We will extract information on abstinence at each time point for which it is reported to allow a survival model to be estimated following the approach used by Chen*, et al.* [30], Madan*, et al.* [31]. Data on reductions in smoking, rather than abstinence, will not be included. Secondary outcomes will include reduction in craving and reduction in withdrawal symptoms.

## Search strategy

We will search the following databases: MEDLINE; EMBASE; PsycINFO; Web of Science; Clinicaltrials.gov; and the Cochrane Databases including the Cochrane Database of Systematic Reviews (CDSR), the Database of Abstracts and Reviews of Effectiveness (DARE) (updated until March 2015), the Cochrane Central Register of Controlled Trials (CENTRAL), the NHS Economic Evaluation Database (NHS EED) and the Health Technology Assessment Database (HTA). Searches will be conducted with the help of an information specialist and will not include any language restrictions. We will also manually search the reference lists of relevant research articles and previous reviews and communicate with authors in an attempt to identify unpublished information; in a previous study we had a 75% positive response rate from corresponding authors for studies published after 2006 [13]. We will also review the literature to identify disutilities and costs associated with neuropsychiatric and cardiovascular treatment-related adverse events. Acknowledging that we may not find many studies in a smoking cessation



For peer review only - http://bmjopen.bmj.com/site/about/guidelines.xhtml

population, we will also search for studies reporting disutilities and costs for the same events in other populations.

To identify studies for the safety network meta-analyses we will build on the basic search strategy included in the cardiovascular network meta-analysis by Mills*, et al.* [32]. Online supplementary appendix 2 presents our full electronic search strategy for observational studies of varenicline in Medline. To identify studies for the effectiveness network meta-analyses the search strategies from four Cochrane reviews [33-36] (or updated versions where available) will be modified to identify more recent trials for inclusion in the current study in addition to their previously identified trials. We will adapt the Sheffield economic model [28] to incorporate disutilities associated with neuropsychiatric and cardiovascular treatment-related adverse events, and update model inputs. The searches used for the network-meta-analyses will be re-run with a cost-effectiveness filter to identify studies reporting information on utilities, disutilities, resource use, and costs, which will be used to inform the economic evaluation.

## Study selection and data extraction

Search results will be uploaded to Covidence [37], which we will use to screen abstracts and full text and to resolve disagreements. Reviewers (MND – all papers, KHT and DC – papers equally shared) will independently screen abstracts to determine whether full text reports should be obtained. The same reviewers will independently identify eligible full text reports for inclusion. Discrepancies will be resolved by reaching consensus amongst reviewers. We will include a PRISMA diagram [38] to set out the results of the searches and to indicate the number of included and excluded trials. The reasons for excluding studies following full text screening will be documented.

Data for studies included following the updated search will be extracted by one reviewer (MND) and checked by co-reviewers (KHT and DC). Information will be collected on: study design (duration of treatment, description of allocation concealment and blinding); study participants (inclusion and exclusion criteria, country, region, population studied); baseline characteristics (e.g., ethnicity, sex, smoking history); intervention and comparison groups (including the smoking cessation intervention, whether or not there was co-treatment, dosage and formulation); our predefined primary and secondary outcomes of interest including measures of effectiveness and safety outcomes; losses to follow up; and study sponsor. All of the extracted information will be summarised in tables. In the event of missing data, we will contact authors by email to ask for original data. Authors of all newly identified studies will be contacted to verify the accuracy of the extracted data.

## Risk of bias assessment

For RCTs the Cochrane tool for assessing the risk of bias [39] will be used to determine whether there is high, low, or unclear risk of bias in the following domains: random sequence generation; allocation concealment; blinding of participants and personnel; blinding of outcome assessment; incomplete outcome data; selective outcome reporting; and other sources of bias. For observational studies we will



For peer review only - http://bmjopen.bmj.com/site/about/guidelines.xhtml

consider using the risk of bias in non-randomized studies of interventions (ROBINS-I) tool [40] to determine whether there is low, moderate, serious, or critical risk of bias or no information in the following domains: confounding; participant selection; intervention classification; intervention deviations; missing data; outcome measurement; and selection of the reported result. Reviewers (MND – all papers, KHT and DC – papers equally shared) will independently assess the risk of bias in each of the trials. Discrepancies will be resolved by referring to the original publication and reaching consensus amongst reviewers. Study authors will be contacted to obtain study protocols and additional information that may not have been published to aid with assessment of the risk of bias.

## Data synthesis

Effectiveness outcomes will be summarised as risk ratios. Because safety outcomes are rare, we will follow recommendations of Bradburn*, et al.* [41] and use Peto odds ratios to compare treatment groups although we will also report risk differences. All results will be reported including 95% confidence intervals. The pairwise and network meta-analyses will be conducted in a Bayesian framework using OpenBUGS software, using code developed by Dias*, et al.* [42]. We will report results of both fixed- and random-effects meta-analyses; between-study heterogeneity will be quantified using the between-study standard deviation ($\tau$). For each separate outcome, we will also construct a network meta-analysis (NMA) that will be compared to the pair-wise results. NMA is an extension of standard meta-analysis that allows evidence synthesis to be performed when there are multiple competing interventions available by simultaneously combining evidence from *all* trials reporting that outcome, so long as treatments form a connected network [43]. Figure 1 shows an example of a connected network of 7 smoking cessation treatments adapted from a Cochrane overview of reviews of smoking cessation at 6 months [10]. For the NMA combination therapies will be considered as separate interventions but we will explore models for the effects of the component therapies using a main effects model and a 2-way interaction model (allowing pairs of therapies to have either a bigger or smaller effect than would be expected from the sum of their effects alone). For the random effects NMA we will assume homogeneous between-study variability across studies. We will assess the goodness of fit of each model to the data by calculating the posterior mean residual deviance. This is defined as the difference between the deviance for the fitted model and the saturated model, where the deviance measures the fit of the model using the likelihood function. The Deviance Information Criterion (DIC), which is equal to the sum of the posterior mean of the residual deviance and the effective number of parameters pD, will be used as a basis for model comparison [44]. The DIC penalises the posterior mean residual deviance (a measure of model fit) by the effective number of parameters in the model (as measure of complexity) and can therefore be viewed as a trade-off between the fit and complexity of the model.

Figure 1. Network diagram for effectiveness of smoking cessation treatments. Adapted from [10].



For peer review only - http://bmjopen.bmj.com/site/about/guidelines.xhtml

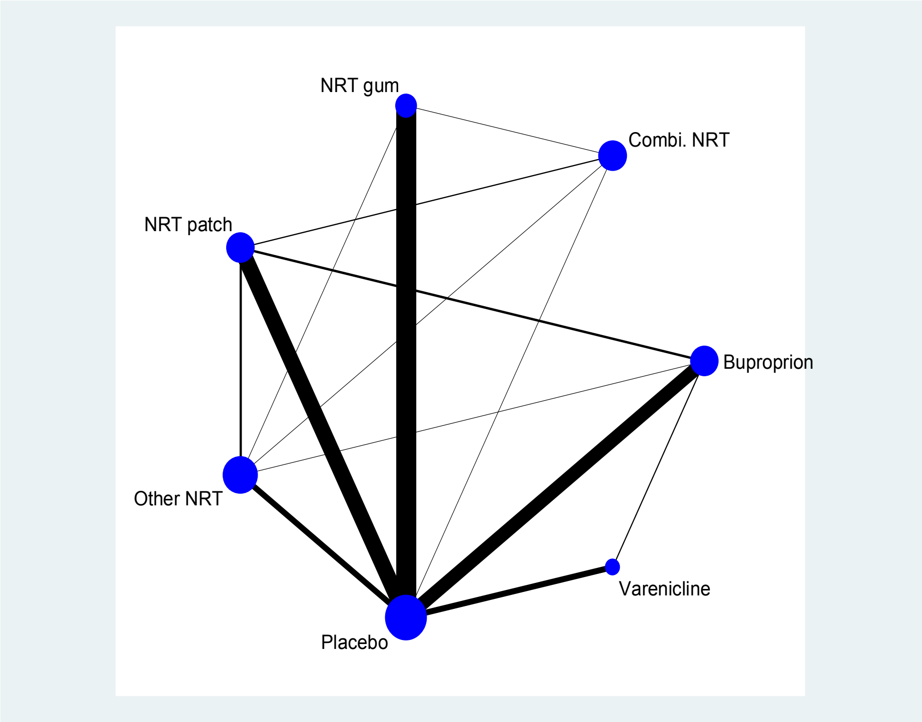


*Note.* The circles represent treatment “nodes” and the solid lines denote the presence of direct trial evidence comparing two treatments. Line thickness is proportional to the number of trials; the thicker the line, the more trials have compared treatments.

For the pairwise analyses only, and where the number of studies allow, we will produce funnel plots.

### Assessment of the consistency assumption

Validity of a NMA depends on the assumption that there is no effect modification of the pairwise intervention effects or, that the prevalence of effect modifiers is similar in the different studies. This key assumption has been referred to variously as transitivity [45], similarity [46] and consistency [47]. We will compile a table of important trial and patient characteristics and visually inspect the ‘similarity’ of factors we consider likely to modify treatment effect.

We will visually inspect the network diagram to identify the extent of potential inconsistency (the number of loops) and use model fit and selection statistics to informally assess whether it is evident. If inconsistency is suspected we will explore it formally using a “node-splitting” approach [47].

### Health economic modelling

A previously built model [28] will be used to evaluate cost-effectiveness using probabilistic methods and reporting fully incremental analyses that meet the NICE reference case [48]. This model has nine health states including those most commonly related to smoking. Where data allow the model will be adapted to allow more than one attempt at quitting and the addition of MANE and MACE events.



For peer review only - http://bmjopen.bmj.com/site/about/guidelines.xhtml

Subgroup analyses will be presented as appropriate. Sensitivity analyses will be performed including and excluding smokeless tobacco users.

# CONCLUSION

The proposed systematic review, network meta-analysis and cost-effectiveness analysis will address important questions about the relative costs or risks and benefits of different smoking cessation pharmacotherapies. It is expected that the study findings will be incorporated into future updates of the new NICE Public Health guidance on Smoking Cessation Interventions and Services, a partial update of the NICE Public Health guidance on Smoking Cessation Services [4], expected to be published in November 2017. Critically, we believe that the study findings will offer patients, prescribers and regulators updated and comprehensive information on the safety and effectiveness of these smoking cessation medicines that will allow them to make informed decisions when evaluating or selecting a pharmacological treatment to assist with a quit attempt.

# ETHICS AND DISSEMINATION

Ethics approval is not required for this evidence synthesis study as it involves analysis of secondary data from randomised controlled trials and observational studies. We anticipate dissemination to the following groups, for whom the results of this research will be of interest: the general public; clinicians and healthcare practitioners; academics; policy makers; and industry. Findings from the study will be disseminated through conventional academic routes such as peer reviewed publications and presentations and regional, national and international conferences.

***Authors' contributions*** KHT, DC and NJW conceived the idea, planned and designed the study protocol. DC designed the network diagram. KHT, DC and NJW planned the data extraction and statistical analyses. NJW and MS planned the economic evaluation. MRM and DG provided critical insight. KHT and MND wrote the first draft of the protocol and all authors approved and contributed to the final manuscript.

**Funding statement** This project was funded by the National Institute for Health Research Health Technology Assessment Programme (project number NIHR HTA 15/58/18). The views and opinions expressed therein are those of the authors and do not necessarily reflect those of the Health Technology Assessment Programme, NIHR, NHS or the Department of Health.

**Competing interests** MRM reports grants from Pfizer, grants from Rusan, and non-financial support from GlaxoSmithKline, outside the submitted work. DG had a specified relationship with the MHRA in the past (was a member of the MHRA's Pharmacovigilance Expert Advisory Group and received travel expenses and a small fee for meeting attendance and preparation for meetings).

# References



For peer review only - http://bmjopen.bmj.com/site/about/guidelines.xhtml

1. Peto R, Darby S, Deo H, Silcocks P, Whitley E, Doll R. Smoking, smoking cessation, and lung cancer in the UK since 1950: combination of national statistics with two case-control studies. BMJ 2000;**321**(7257):323-9
2. Jha P, Peto R. Global Effects of Smoking, of Quitting, and of Taxing Tobacco. N Engl J Med 2014;**370**(1):60-68 doi: doi:10.1056/NEJMra1308383[published Online First: Epub Date]|.
3. Health and Social Care Information Centre. Statistics on Smoking: England, 2012, 2012.
4. National Institute for Health and Clinical Excellence. NICE public health guidance 10. Smoking cessation services in primary care, pharmacies, local authorities and workplaces, particularly for manual working groups, pregnant women and hard to reach communities. London: NICE, 2008.
5. Medicines & Healthcare products Regulatory Agency. Public Assessment Report. e-Voke 10mg and 15mg Electronic Inhaler (PL 42601/0003-4). Secondary Public Assessment Report. e-Voke 10mg and 15mg Electronic Inhaler (PL 42601/0003-4) 2015. http://www.mhra.gov.uk/home/groups/par/documents/websiteresources/con616843.pdf.
6. Medicines and Healthcare products Regulatory Agency. Varenicline:adverse psychiatric reactions, including depression. Drug Safety Update 2008;**2**(4):2-3
7. US Food and Drug Administration. Information for Healthcare Professionals: Varenicline (marketed as Chantix) and Bupropion (marketed as Zyban, Wellbutrin and generics). FDA Drug Safety Newsletter 2009; 2(1). http://www.fda.gov/Drugs/DrugSafety/PostmarketDrugSafetyInformationforPatientsandProviders/DrugSafetyInformationforHeathcareProfessionals/ucm169986.htm.
8. Harrison-Woolrych M. Mental health effects of varenicline. BMJ : British Medical Journal 2015;**350**
9. Action on Smoking and Health. Use of electronic cigarettes (vapourisers) among adults in Great Britain, 2016.
10. Cahill K, Stevens S, Perera R, Lancaster T. Pharmacological interventions for smoking cessation: an overview and network meta-analysis. Cochrane Database Syst Rev 2013;**5** doi: 10.1002/14651858.CD009329.pub2[published Online First: Epub Date]|.
11. Prescribing and Primary Care team Health and Social Care Information Centre. Prescription Cost Analysis, England, 2013. 2014. http://www.hscic.gov.uk/catalogue/PUB13887/pres-cost-anal-eng-2013-rep.pdf.
12. Tonstad S, Davies S, Flammer M, Russ C, Hughes J. Psychiatric Adverse Events in Randomized, Double-Blind, Placebo-Controlled Clinical Trials of Varenicline. Drug-Safety 2010;**33**(4):289-301 doi: 10.2165/11319180-000000000-00000[published Online First: Epub Date]|.
13. Thomas KH, Martin RM, Knipe DW, Higgins JP, Gunnell D. Risk of neuropsychiatric adverse events associated with varenicline: systematic review and meta-analysis. BMJ 2015;**350**:h1109 doi: 10.1136/bmj.h1109[published Online First: Epub Date]|.
14. Gibbons RD, Mann JJ. Varenicline, Smoking Cessation, and Neuropsychiatric Adverse Events. The American journal of psychiatry 2013 doi: 10.1176/appi.ajp.2013.12121599[published Online First: Epub Date]|.
15. Moore TJ, Furberg CD, Glenmullen J, Maltsberger JT, Singh S. Suicidal Behavior and Depression in Smoking Cessation Treatments. Plos One 2011;**6**(11):e27016 doi: 10.1371/journal.pone.0027016[published Online First: Epub Date]|.
16. Harrison-Woolrych M, Ashton J. Psychiatric Adverse Events Associated with Varenicline: An Intensive Postmarketing Prospective Cohort Study in New Zealand. Drug Saf 2011;**34**(9):763-72



For peer review only - http://bmjopen.bmj.com/site/about/guidelines.xhtml

17. Kasliwal R, Wilton LV, Shakir SAW. Safety and Drug Utilization Profile of Varenicline as Used in General Practice in England: Interim Results from a Prescription-Event Monitoring Study. Drug Saf 2009;**32**(6):499-507 doi: 10.2165/00002018-200932060-00006[published Online First: Epub Date]|.
18. Thomas KH, Martin RM, Davies N, Metcalfe C, Windmeijer F, Gunnell D. Smoking cessation treatment and the risk of depression, suicide and self-harm in the Clinical Practice Research Datalink: prospective cohort study BMJ 2013;**347**:f5704
19. Gunnell D, Irvine D, Wise L, Davies C, Martin RM. Varenicline and suicidal behaviour: a cohort study based on data from the General Practice Research Database. Brit Med J 2009;**339** doi: Artn B3805 Doi 10.1136/Bmj.B3805[published Online First: Epub Date]|.
20. Meyer TE, Taylor LG, Xie S, et al. Neuropsychiatric events in varenicline and nicotine replacement patch users in the Military Health System. Addiction 2013;**108**(1):203-10 doi: 10.1111/j.1360-0443.2012.04024.x[published Online First: Epub Date]|.
21. Campbell AR, Anderson KD. Mental health stability in veterans with posttraumatic stress disorder receiving varenicline. American journal of health-system pharmacy : AJHP : official journal of the American Society of Health-System Pharmacists 2010;**67**(21):1832-7 doi: 10.2146/ajhp100196[published Online First: Epub Date]|.
22. Thomas KH, Martin RM, Potokar J, Pirmohamed M, Gunnell D. Reporting of drug induced depression and fatal and non-fatal suicidal behaviour in the UK from 1998 to 2011. BMC Pharmacology and Toxicology 2014;**15**(1):1-11 doi: 10.1186/2050-6511-15-54[published Online First: Epub Date]|.
23. Davis S, King B, Raine JM. Spontaneous reporting- UK. In: Mann RD, Andrews EB, eds. Pharmacovigilance: Second Edition Chichester, West Sussex: John Wiley & Sons, 2007:199-215.
24. Martin RM, May M, Gunnell D. Did intense adverse media publicity impact on prescribing of paroxetine and the notification of suspected adverse drug reactions? Analysis of routine databases, 2001-2004. Br J Clin Pharmacol 2006;**61**:224-28
25. Walker AM. Confounding by indication. Epidemiology (Cambridge, Mass.) 1996;**7**(4):335-6
26. Kotz D, Viechtbauer W, Simpson C, van Schayck OCP, West R, Sheikh A. Cardiovascular and neuropsychiatric risks of varenicline: a retrospective cohort study. The Lancet Respiratory Medicine 2015;**3**(10):761-68 doi: http://dx.doi.org/10.1016/S2213-2600(15)00320-3[published Online First: Epub Date]|.
27. US Department of Health and Human Services. The Health Consequences of Smoking-50 Years of Progress: A Report of the Surgeon General. Atlanta (GA): US Department of Health and Human Services, Centers for Disease Control and Prevention, National Center for Chronic Disease Prevention, Health Promotion Office on Smoking Health, 2014.
28. Leaviss J, Sullivan W, Ren S, et al. What is the clinical effectiveness and cost-effectiveness of cytisine compared with varenicline for smoking cessation? A systematic review and economic evaluation. Health technology assessment (Winchester, England) 2014;**18**(33):1-120 doi: 10.3310/hta18330[published Online First: Epub Date]|.
29. Hertzberg MA, Moore SD, Feldman ME, Beckham JC. A preliminary study of bupropion sustained-release for smoking cessation in patients with chronic posttraumatic stress disorder. Journal of clinical psychopharmacology 2001;**21**(1):94-8
30. Chen YF, Madan J, Welton N, et al. Effectiveness and cost-effectiveness of computer and other electronic aids for smoking cessation: a systematic review and network meta-



For peer review only - http://bmjopen.bmj.com/site/about/guidelines.xhtml

analysis. Health technology assessment (Winchester, England) 2012;**16**(38):1-205, iii-v doi: 10.3310/hta16380[published Online First: Epub Date]|.
31. Madan J, Chen Y-F, Aveyard P, et al. Synthesis of evidence on heterogeneous interventions with multiple outcomes recorded over multiple follow-up times reported inconsistently: a smoking cessation case-study. Journal of the Royal Statistical Society: Series A (Statistics in Society) 2014;**177**(1):295-314 doi: 10.1111/rssa.12018[published Online First: Epub Date]|.
32. Mills EJ, Thorlund K, Eapen S, Wu P, Prochaska JJ. Cardiovascular events associated with smoking cessation pharmacotherapies: a network meta-analysis. Circulation 2014;**129**(1):28-41 doi: 10.1161/circulationaha.113.003961[published Online First: Epub Date]|.
33. Cahill K, Lindson-Hawley N, Thomas KH, Fanshawe TR, Lancaster T. Nicotine receptor partial agonists for smoking cessation. Cochrane Database of Systematic Reviews 2016(5) doi: 10.1002/14651858.CD006103.pub7[published Online First: Epub Date]|.
34. Hughes J, R., Stead LF, Hartmann-Boyce J, Cahill K, Lancaster T. Antidepressants for smoking cessation. Cochrane Database of Systematic Reviews 2014(1) doi: 10.1002/14651858.CD000031.pub4[published Online First: Epub Date]|.
35. Stead LF, Perera R, Bullen C, et al. Nicotine replacement therapy for smoking cessation. Cochrane database of systematic reviews (Online) 2012;**11**
36. Hartmann-Boyce J, McRobbie H, Bullen C, Begh R, Stead LF, Hajek P. Electronic cigarettes for smoking cessation. Cochrane Database of Systematic Reviews 2016(9) doi: 10.1002/14651858.CD010216.pub3[published Online First: Epub Date]|.
37. Covidence systematic review software. Melbourne, Australia: Veritas Health Innovation. Available at http://www.covidence.org/
38. Moher D, Liberati A, Tetzlaff J, Altman DG. Preferred reporting items for systematic reviews and meta-analyses: the PRISMA statement. BMJ 2009;**339** doi: 10.1136/bmj.b2535[published Online First: Epub Date]|.
39. Higgins JPT, Altman DG, Gøtzsche PC, et al. The Cochrane Collaboration's tool for assessing risk of bias in randomised trials. BMJ 2011;**343**
40. Sterne JAC, Hernán MA, Reeves BC, et al. ROBINS-I: a tool for assessing risk of bias in non-randomised studies of interventions. BMJ 2016;**355**
41. Bradburn MJ, Deeks JJ, Berlin JA, Russell Localio A. Much ado about nothing: a comparison of the performance of meta-analytical methods with rare events. Statistics in medicine 2007;**26**(1):53-77 doi: 10.1002/sim.2528[published Online First: Epub Date]|.
42. Dias S, Sutton AJ, Ades AE, Welton NJ. Evidence Synthesis for Decision Making 2: A Generalized Linear Modeling Framework for Pairwise and Network Meta-analysis of Randomized Controlled Trials. Medical Decision Making 2013;**33**(5):607-17 doi: 10.1177/0272989x12458724[published Online First: Epub Date]|.
43. Caldwell DM, Ades AE, Higgins JPT. Simultaneous comparison of multiple treatments: combining direct and indirect evidence. BMJ 2005;**331**(7521):897
44. Spiegelhalter DJ, Best NG, Carlin BP, Van Der Linde A. Bayesian measures of model complexity and fit. Journal of the Royal Statistical Society: Series B (Statistical Methodology) 2002;**64**(4):583-639 doi: 10.1111/1467-9868.00353[published Online First: Epub Date]|.
45. Salanti G, Higgins JP, Ades A, Ioannidis JP. Evaluation of networks of randomized trials. Statistical Methods in Medical Research 2008;**17**(3):279-301 doi: 10.1177/0962280207080643[published Online First: Epub Date]|.
46. Song F, Altman DG, Glenny AM, Deeks JJ. Validity of indirect comparison for estimating efficacy of competing interventions: empirical evidence from published



For peer review only - http://bmjopen.bmj.com/site/about/guidelines.xhtml

meta-analyses. Bmj 2003;**326**(7387):472 doi: 10.1136/bmj.326.7387.472[published Online First: Epub Date]|.

47. Dias S, Welton NJ, Caldwell DM, Ades AE. Checking consistency in mixed treatment comparison meta-analysis. Statistics in medicine 2010;**29**(7-8):932-44 doi: 10.1002/sim.3767[published Online First: Epub Date]|.

48. National Institute for Health and Clinical Excellence. Guide to the Methods of Technology Appraisal 2013. London: NICE, 2013.

For peer review only



For peer review only - http://bmjopen.bmj.com/site/about/guidelines.xhtml

Appendix 1. Summary of pharmacologic interventions by dose and formulation.

| Treatment and formulation | Lower dose | Standard dose | Higher dose |
|---|---|---|---|
| *Bupropion* | | | |
| Oral extended release tablets | <150 mg bd | 150 mg bd | >150 mg bd |
| *Varenicline* | | | |
| Tablets | <1 mg bd | 1 mg bd | >1 mg bd |
| *Nicotine replacement therapy* | | | |
| Patch (16 hrs) | <15 mg (5 mg/16 hours or 10 mg/16 hours) | 15 mg (15 mg/16 hours) | >15 mg (25 mg/16 hours) |
| Patch (24 hrs) | < 14 mg (7 mg/24 hours) | 14 mg (14 mg/24 hours) | >14 mg (21 mg/24 hours) |
| Gum | | 2 mg/piece (15 pieces daily) | 4 mg/piece (15 pieces daily) |
| Nasal spray | | 0.5 mg/metered spray (up to 2 sprays/hour, 64 daily) | |
| Mouth spray | | 1 mg/metered spray (up to 4 sprays/hour, 64 daily) | |
| Lozenge | 1 mg or 1.5 mg (1 lozenge/1-2 hours, 15 daily) | 2 mg (1 lozenge/1-2 hours, 15 daily) | 4 mg (1 lozenge/1-2 hours, 15 daily) |
| Sublingual tablet | | 2 mg per tablet (up to 1 tablet/hour, 40 daily) | 2 mg per tablet (up to 2 tablets/hour, 40 daily) |
| Inhalator | | 10 mg/cartridge (12 cartridges daily) | 15 mg/cartridge (6 cartridges daily) |
| *Electronic Cigarette* | | | |
| Electronic Inhaler | | 10mg/cartridge (5 cartridges daily) | 15mg/cartridge (5 cartridges daily) |

*Note*. Maximum doses in parentheses; mg = milligram; bd = twice daily. For nicotine replacement therapy the higher dose is generally used in smokers who smoke >20 cigarettes per day.

For peer review only

Appendix 2. Full electronic search strategy for observational studies of varenicline in Medline.

1 Smoking/

2 Tobacco/

3 Nicotine/

4 Tobacco Products/

5 Smoking/dt [Drug Therapy]

6 Smoking Cessation/

7 "Tobacco Use Cessation"/

8 "Tobacco Use Disorder"/

9 Smoking/pc [Prevention & Control]

10 (smoking or smoker*).ti,ab,kf.

11 (tobacco* or cigar* or cigarette* or nicotine).ti,ab,kf.

12 ((smoking or tobacco) adj5 (cessation or ceas* or quit* or stop* or giv* or prevent* or abstain* or abstin* or control*)).ti,ab,kf.

13 Varenicline/

14 Nicotinic Agonists/

15 varenicline.ti,ab,kf.

16 (champix or tabex or chantix).ti,ab,kf.

17 (nicotinic adj3 agonist*).ti,ab,kf.

18 (benzazepine* adj2 derivative*).ti,ab,kf.

19 nicotinic receptor partial agonist*.ti,ab,kf.

20 Epidemiologic Methods/ (30872)

21 epidemiologic studies/

22 case-control studies/ or retrospective studies/

23 cohort studies/ or follow-up studies/ or longitudinal studies/ or "national longitudinal study of adolescent health"/ or prospective studies/ or retrospective studies/

24 case-control studies/

25 Cross-Sectional Studies/

26 case control.ti,ab,kf.

27 (cohort adj (study or studies)).ti,ab,kf.



For peer review only - http://bmjopen.bmj.com/site/about/guidelines.xhtml

| | |
|---|---|
| 28 | cohort analy*.ti,ab,kf. |
| 29 | (follow-up adj (study or studies)).ti,ab,kf. |
| 30 | (observational adj (study or studies)).ti,ab,kf. |
| 31 | (longitudinal or retrospective or prospective or cross sectional).ti,ab,kf. |
| 32 | (epidemiologic* adj (study or studies)).ti,ab,kf. |
| 33 | 1 or 2 or 3 or 4 or 5 or 6 or 7 or 8 or 9 or 10 or 11 or 12 |
| 34 | 13 or 14 or 15 or 16 or 17 or 18 or 19 |
| 35 | 20 or 21 or 22 or 23 or 24 or 25 or 26 or 27 or 28 or 29 or 30 or 31 or 32 |
| 36 | 33 and 34 and 35 |

For peer review only



For peer review only - http://bmjopen.bmj.com/site/about/guidelines.xhtml

# PRISMA 2009 Checklist

| Section/topic | # | Checklist item | Reported on page # |
|---|---|---|---|
| **TITLE** | | | |
| Title | 1 | Identify the report as a systematic review, meta-analysis, or both. | 1 |
| **ABSTRACT** | | | |
| Structured summary | 2 | Provide a structured summary including, as applicable: background; objectives; data sources; study eligibility criteria, participants, and interventions; study appraisal and synthesis methods; results; limitations; conclusions and implications of key findings; systematic review registration number. | 1-2 |
| **INTRODUCTION** | | | |
| Rationale | 3 | Describe the rationale for the review in the context of what is already known. | 3-5 |
| Objectives | 4 | Provide an explicit statement of questions being addressed with reference to participants, interventions, comparisons, outcomes, and study design (PICOS). | 3-6 |
| **METHODS** | | | |
| Protocol and registration | 5 | Indicate if a review protocol exists, if and where it can be accessed (e.g., Web address), and, if available, provide registration information including registration number. | N/A |
| Eligibility criteria | 6 | Specify study characteristics (e.g., PICOS, length of follow-up) and report characteristics (e.g., years considered, language, publication status) used as criteria for eligibility, giving rationale. | 5-6 |
| Information sources | 7 | Describe all information sources (e.g., databases with dates of coverage, contact with study authors to identify additional studies) in the search and date last searched. | 6-7 |
| Search | 8 | Present full electronic search strategy for at least one database, including any limits used, such that it could be repeated. | 16 |
| Study selection | 9 | State the process for selecting studies (i.e., screening, eligibility, included in systematic review, and, if applicable, included in the meta-analysis). | 7 |
| Data collection process | 10 | Describe method of data extraction from reports (e.g., piloted forms, independently, in duplicate) and any processes for obtaining and confirming data from investigators. | 7-8 |
| Data items | 11 | List and define all variables for which data were sought (e.g., PICOS, funding sources) and any assumptions and simplifications made. | 7 |
| Risk of bias in individual studies | 12 | Describe methods used for assessing risk of bias of individual studies (including specification of whether this was done at the study or outcome level), and how this information is to be used in any data synthesis. | 8 |
| Summary measures | 13 | State the principal summary measures (e.g., risk ratio, difference in means). | 8 |
| Synthesis of results | 14 | Describe the methods of handling data and combining results of studies, if done, including measures of consistency (e.g., $I^2$) for each meta-analysis. | 8-9 |

For peer review only - http://bmjopen.bmj.com/site/about/guidelines.xhtml

# PRISMA 2009 Checklist



| Section/topic | # | Checklist item | Reported on page # |
|---|---|---|---|
| Risk of bias across studies | 15 | Specify any assessment of risk of bias that may affect the cumulative evidence (e.g., publication bias, selective reporting within studies). | 9 |
| Additional analyses | 16 | Describe methods of additional analyses (e.g., sensitivity or subgroup analyses, meta-regression), if done, indicating which were pre-specified. | 10 |
| **RESULTS** | | | |
| Study selection | 17 | Give numbers of studies screened, assessed for eligibility, and included in the review, with reasons for exclusions at each stage, ideally with a flow diagram. | N/A |
| Study characteristics | 18 | For each study, present characteristics for which data were extracted (e.g., study size, PICOS, follow-up period) and provide the citations. | N/A |
| Risk of bias within studies | 19 | Present data on risk of bias of each study and, if available, any outcome level assessment (see item 12). | N/A |
| Results of individual studies | 20 | For all outcomes considered (benefits or harms), present, for each study: (a) simple summary data for each intervention group (b) effect estimates and confidence intervals, ideally with a forest plot. | N/A |
| Synthesis of results | 21 | Present results of each meta-analysis done, including confidence intervals and measures of consistency. | N/A |
| Risk of bias across studies | 22 | Present results of any assessment of risk of bias across studies (see Item 15). | N/A |
| Additional analysis | 23 | Give results of additional analyses, if done (e.g., sensitivity or subgroup analyses, meta-regression [see Item 16]). | N/A |
| **DISCUSSION** | | | |
| Summary of evidence | 24 | Summarize the main findings including the strength of evidence for each main outcome; consider their relevance to key groups (e.g., healthcare providers, users, and policy makers). | N/A |
| Limitations | 25 | Discuss limitations at study and outcome level (e.g., risk of bias), and at review-level (e.g., incomplete retrieval of identified research, reporting bias). | N/A |
| Conclusions | 26 | Provide a general interpretation of the results in the context of other evidence, and implications for future research. | 10 |
| **FUNDING** | | | |
| Funding | 27 | Describe sources of funding for the systematic review and other support (e.g., supply of data); role of funders for the systematic review. | 10 |

*From:* Moher D, Liberati A, Tetzlaff J, Altman DG, The PRISMA Group (2009). Preferred Reporting Items for Systematic Reviews and Meta-Analyses: The PRISMA Statement. PLoS Med 6(6): e1000097. doi:10.1371/journal.pmed1000097

For more information, visit: **www.prisma-statement.org**.



For peer review only - http://bmjopen.bmj.com/site/about/guidelines.xhtml

BMJ Open

# How do smoking cessation medicines compare with respect to their neuropsychiatric safety? A protocol for a systematic review, network meta-analysis and cost-effectiveness analysis


| | |
|---|---|
| Journal: | *BMJ Open* |
| Manuscript ID | bmjopen-2016-015414.R1 |
| Article Type: | Protocol |
| Date Submitted by the Author: | 10-Mar-2017 |



| | |
|---|---|
| Complete List of Authors: | Thomas, Kyla; University of Bristol, School of Social and Community Medicine<br>Caldwell, Deborah; University of Bristol, School of Social and Community Medicine<br>Dalili, Michael; University of Bristol, School of Social and Community Medicine<br>Gunnell, DJ; University of Bristol, School of Social and Community Medicine<br>Munafò, Marcus; University of Bristol, MRC Integrative Epidemiology Unit; University of Bristol, UK Centre for Tobacco and Alcohol Studies, School of Experimental Psychology<br>Stevenson, Matt; University of Sheffield School of Health and Related Research<br>Welton, Nicky; University of Bristol, School of Social and Community Medicine |



| | |
|---|---|
| <b>Primary Subject Heading</b>: | Smoking and tobacco |
| Secondary Subject Heading: | Public health, Pharmacology and therapeutics |
| Keywords: | varenicline, bupropion, nicotine replacement therapy, electronic cigarettes, smoking cessation, network meta-analysis |


SCHOLARONE™
Manuscripts

# *How do smoking cessation medicines compare with respect to their neuropsychiatric safety? A protocol for a systematic review, network meta-analysis and cost-effectiveness analysis*

Kyla H Thomas[1], Deborah Caldwell[1], Michael N Dalili[1], David Gunnell[1], Marcus R Munafò[2,3], Matt Stevenson[4], Nicky J Welton[1]

1 School of Social and Community Medicine, University of Bristol, Bristol, UK

2 MRC Integrative Epidemiology Unit, University of Bristol, Bristol, UK

3 UK Centre for Tobacco and Alcohol Studies, School of Experimental Psychology, University of Bristol, Bristol, UK

4 School of Health and Related Research, University of Sheffield, Sheffield, UK

Correspondence to
Dr Kyla H Thomas; School of Social and Community Medicine, University of Bristol, Canynge Hall, Room 3.12, 39 Whatley Road, Bristol, United Kingdom BS8 2PS
kyla.thomas@bristol.ac.uk

Dr Deborah Caldwell, School of Social and Community Medicine, University of Bristol, Bristol, UK d.m.caldwell@bristol.ac.uk

Dr Michael Dalili, School of Social and Community Medicine, University of Bristol, Bristol, UK michael.dalili@bristol.ac.uk

Professor David Gunnell, School of Social and Community Medicine, University of Bristol, Bristol, UK d.j.gunnell@bristol.ac.uk

Professor Marcus Munafò, MRC Integrative Epidemiology Unit, University of Bristol, Bristol, UK; UK Centre for Tobacco and Alcohol Studies, School of Experimental Psychology, University of Bristol, Bristol, UK marcus.munafo@bristol.ac.uk

Professor Matt Stevenson, School of Health and Related Research, University of Sheffield, Sheffield, UK m.d.stevenson@sheffield.ac.uk

Dr Nicky Welton, School of Social and Community Medicine, University of Bristol, Bristol, UK nicky.welton@bristol.ac.uk

# ABSTRACT

### Introduction

Cigarette smoking is one of the leading causes of early death in the UK and worldwide. Public health guidance recommends the use of varenicline, bupropion and nicotine replacement therapy (NRT) as smoking cessation aids in the UK. Additionally, the first electronic cigarette has been licensed for use as a smoking



For peer review only - http://bmjopen.bmj.com/site/about/guidelines.xhtml

For peer review only

cessation medicine. However, there are ongoing concerns about the safety of these medicines. We present a protocol for a systematic review and network meta-analysis (NMA) to determine how these smoking cessation medicines compare to each other with respect to their neuropsychiatric safety in adult smokers. Secondary aims include updating the evidence regarding the effectiveness and cardiovascular safety of these medicines for use in a cost-effectiveness analysis.

**Methods and analysis**

We will include randomised controlled trials and observational studies with control groups comparing monotherapy with varenicline, bupropion, NRT or electronic cigarette and combination therapies to each other, placebo or usual care. The primary composite safety outcome will be serious adverse events, defined as events that resulted in death, were life threatening, required hospitalisation, or resulted in significant disability or congenital/birth defect. The preferred effectiveness outcome will be sustained smoking cessation defined as abstinence for a minimum of six months as determined by biochemical validation. We will include trials identified by previous reviews and search relevant databases for newly published trials as well as contacting study authors to identify unpublished information. We will conduct fixed- and random-effects meta-analyses for each pairwise comparison of treatments and outcome; where these estimates differ we will consider reasons for heterogeneity, quantified using the between-study variance ($\tau^2$). For each outcome, we will construct a NMA in a Bayesian framework which will be compared to the pair-wise results, allowing us to rank treatments. The effectiveness estimates from the NMA will be entered into a probabilistic economic model.

**Ethics and dissemination**

Ethics approval is not required for this evidence synthesis study as it involves analysis of secondary data from randomised controlled trials and observational studies. The review will make an important contribution to the knowledge base around the effectiveness, safety and cost-effectiveness of smoking cessation medicines. Results will be disseminated to the general public, healthcare practitioners and clinicians, academics, industry and policy makers.

**PROSPERO registration number** CRD42016041302

For peer review only

# STRENGTHS AND LIMITATIONS OF THIS STUDY

- This study will be the first comprehensive review of the neuropsychiatric safety of smoking cessation medicines in relation to each other and will include randomised controlled trials of any duration as well as observational studies with control groups. Including observational studies will allow us to identify sufficient data for the NMA and improve the precision of estimates of adverse events.

- This study will include trials of combined therapies of smoking cessation medicines, not included in previous network meta-analyses, as the analysis



For peer review only - http://bmjopen.bmj.com/site/about/guidelines.xhtml

of both safety and efficacy data on co-prescribing could be of importance to regulators.

- This study will produce updated cost-effectiveness analyses to estimate which smoking cessation medicine is the most cost-effective in UK settings.
- We will use the Cochrane tool for assessing the risk of bias and consider using the risk of bias in non-randomized studies of interventions (ROBINS-I) tool to assess the risk of bias in randomised controlled trials and observational studies, respectively.
- This study will be limited to products licensed for use as smoking cessation aids in the UK.

# INTRODUCTION

Cigarette smoking is one of the leading causes of early death in the UK and worldwide [1,2]. Each year, more than 100,000 people will die in the UK from smoking related diseases [3]. NICE public health guidance (published in 2008) recommends the use of three medicines, varenicline, bupropion and nicotine replacement therapy (NRT), as aids to quitting smoking in the UK [4]. Additionally, in late 2015 the Medicines and Healthcare Products Regulatory Agency (MHRA) approved the use of British American Tobacco's electronic cigarette "e-Voke" as a smoking cessation medicine [5]. Since the publication of the original NICE guidance, there have been ongoing concerns about the safety of the smoking cessation medicines, with particular respect to the neuropsychiatric safety of varenicline. Severe safety warnings (black triangle and black box warnings) regarding a potential increased risk of serious neuropsychiatric adverse events (depression, suicidal ideation and suicidal behaviour) in patients prescribed these medicines have previously been issued by regulatory agencies such as the MHRA in the UK and the Food and Drug Administration (FDA) in the US [6,7]. These safety warnings were based on spontaneous reports to the UK Yellow Card Scheme and the FDA Adverse Events Reporting Database. Previous research into the neuropsychiatric safety of these medicines has provided inconsistent findings, adding to the debate [8]. In April 2016, the results of EAGLES trial [9], a randomised controlled trial (RCT) that randomised 8144 smokers to receive varenicline, transdermal NRT patch, bupropion or placebo, were published. The trial's findings provided strong evidence that both varenicline and bupropion do not cause an increase in neuropsychiatric adverse events relative to nicotine patch or placebo. Subsequently, the European Medicines Agency (EMA) lifted the warning about possible suicidal risks from varenicline in April 2016 [10], followed by the FDA's decision to remove the black box warnings on varenicline's labelling in December 2016. While the EAGLES trial may be the largest global clinical trial of smoking cessation medicines, its sample size is limited relative to much larger observational cohort studies [11,12]. Therefore, synthesising these findings with those of other RCTs, as well as synthesising the results of observational studies, will offer a more comprehensive review of the neuropsychiatric

For peer review only



For peer review only - http://bmjopen.bmj.com/site/about/guidelines.xhtml

safety of smoking cessation medicines. With the MHRA's licensing of the first electronic cigarette as a smoking cessation medicine, and given the popularity of electronic cigarettes with an estimated 2.8 million adult users in Great Britain [13], it is important to review their effectiveness as a smoking cessation aid as well as their safety.

The health benefits of smoking cessation have been well documented. Varenicline has been shown to be the most clinically effective monotherapy for long-term smoking abstinence (>6 months) [14]. However, the number of prescription items of varenicline dispensed in England decreased by 25% from a peak of approximately one million prescriptions in 2011 to almost 742,000 prescriptions in 2013 [15], possibly reflecting ongoing fears among prescribers and patients regarding varenicline's neuropsychiatric safety. It is important for patients, prescribers and regulators to know how smoking cessation medicines compare with each other, with particular respect to their neuropsychiatric safety, to enable smokers wanting to quit and their healthcare professionals to make informed decisions about the risks and benefits of the different pharmacological treatments. To date, there has been no comprehensive analysis of the neuropsychiatric safety of the smoking cessation medicines; previous systematic reviews have focussed on comparisons of varenicline with placebo [16-18]. In addition, the cost-effectiveness of these medicines in UK settings has not been investigated using the most up to date evidence. These analyses are important to inform the overall risk-benefit evaluation of the different smoking cessation medicines.

The ongoing debate regarding the neuropsychiatric safety of drugs for smoking cessation among drug regulators, researchers, prescribers and patients may be due to the inconsistent research findings in this area [8]. Whereas studies without control groups (such as those using adverse event reporting data and case studies) [19-21] have reported increased risks of self-reported depression and suicidal or self-injurious behaviour in patients prescribed varenicline and bupropion, studies with control groups (such as observational cohort studies and experimental study designs, mainly RCTs and systematic reviews of RCTs) have reported the opposite, and found no evidence of an increased risk of these severe neuropsychiatric outcomes in patients prescribed these medicines [11,16-18,22-24]. However, there are important limitations associated with each of these study designs. First, studies which use spontaneous adverse event reports are limited by several factors. These include the severity of the adverse event (severe adverse events are more likely to be reported than less serious events) [25], the length of time that the drug has been available (adverse events with newer drugs are more likely to be reported than events occurring with older drugs for the same indication) and media publicity about a drug (media reports often lead to increased reporting of adverse events to the Yellow Card Scheme, known as stimulated reporting) [26,27]. Second, observational cohort studies are prone to the effects of confounding by indication, which raises concerns about the validity of their findings [28]. Confounding by indication may occur if an observed association between smoking cessation medicines and serious psychiatric events such as suicide is explained if the patient case mix differs among those taking different smoking cessation medications [11,12,29]. In two of the earlier


For peer review only - http://bmjopen.bmj.com/site/about/guidelines.xhtml

cohort studies, varenicline and bupropion were found to be associated with a decreased risk of death from all causes compared to NRT [11,22]; this reported protective effect was most likely due to residual confounding in the studies.

Experimental studies are less likely to suffer from uncontrolled confounding, however, to date, systematic reviews and meta-analyses of RCTs have mainly focussed on comparing the neuropsychiatric safety of varenicline monotherapy with placebo [16-18,30]. Although this is an important research question, patients are unlikely to be prescribed placebo in real life settings to help them quit smoking. Therefore the neuropsychiatric safety of varenicline compared to other smoking cessation drugs is likely to be of greater relevance to patients, prescribers and regulators. To date, there have been no comprehensive reviews of the neuropsychiatric safety of the smoking cessation medicines in relation to each other. In addition, there have been no recent cost-effectiveness analyses that have fully accounted for adverse events in order to determine which UK-licensed smoking cessation medicine is estimated to be the most cost-effective in UK settings. The aim of this study is to determine how smoking cessation medicines compare to each other with respect to their neuropsychiatric safety in adult smokers. Secondary aims include updating the evidence regarding the effectiveness and cardiovascular safety of these medicines for use in a cost-effectiveness analysis.

# METHODS AND ANALYSIS

## Criteria for screening studies

### Study designs

For the safety network meta-analyses, RCTs of any duration will be included in addition to observational studies with control groups. Uncontrolled observational studies (e.g., case reports and case series) will be excluded.

For the effectiveness network meta-analyses, we will include RCTs of greater than 6 months' duration in any setting, including, but not limited to, primary care practices, hospitals including inpatient and outpatient clinics, universities, workplace clinics, nursing or residential homes. Multi-arm trials will be included while crossover trials, non-randomised trials, quasi-randomised trials and interrupted time series analyses will be excluded from the effectiveness analyses.

### Participants

We will include smokers aged 18 years and over of all ethnicities who are seeking to quit smoking using UK-licensed smoking cessation therapies. This includes adult smokers accessing local authority stop smoking services. We will also include smokeless tobacco users. We will exclude studies with participants less than 18 years of age as varenicline, bupropion and electronic cigarettes are only licensed for use in adults in the UK. Non-smoking populations will be excluded as well as pregnant and breastfeeding women, as varenicline and bupropion are not licensed for use in these groups in the UK. If sufficient evidence is identified, we plan to consider the following subgroups in all analyses: those with psychiatric illness (for



For peer review only - http://bmjopen.bmj.com/site/about/guidelines.xhtml

example depression, schizophrenia, bipolar disorder, substance misuse); cardiovascular disease (for example peripheral vascular disease, acute coronary syndromes and post myocardial infarction); Chronic Obstructive Pulmonary Disease; diabetics; heavy smokers (defined as people who smoke >20 cigarettes per day); and those with previous quit attempts.

## Interventions

The smoking cessation medicines that we will assess include varenicline, bupropion, nicotine replacement therapy and electronic cigarette as monotherapy and in combination treatment (e.g. varenicline combined with NRT, varenicline combined with bupropion and bupropion compared to NRT). For NRT, combinations of different formulations given concurrently, for example patch and gum, will also be assessed. Different dosages of treatments will also be examined. Online supplementary appendix 1 summarises the main pharmacologic monotherapies for smoking cessation by formulation and dosage using the British National Formulary (BNF) September 2016 version (http://www.evidence.nhs.uk/formulary/bnf/current) and the MHRA public assessment report for the “e-Voke” [5]. Combination therapies and combinations of NRT formulations will also be included in the analyses.

We will exclude trials of alternative and complementary therapies (e.g., hypnotherapy, acupuncture, aromatherapy and herbal therapies), and psychotherapies (unless they are included as co-treatment with a pharmacologic intervention).

## Outcomes

## Safety analysis

The primary composite safety outcome will be serious adverse events, defined as events that resulted in death, were life threatening, required hospitalisation, or resulted in significant disability or congenital/birth defect [31]. Safety outcomes will be grouped under the following headings:

- Neuropsychiatric outcomes. The primary neuropsychiatric outcomes will include: completed suicide; attempted suicide; suicidal ideation; depression; and seizures. Secondary neuropsychiatric outcomes will include: abnormal dreams; aggression; anxiety; fatigue; insomnia; irritability; sleep disorders; and somnolence.
- Cardiovascular outcomes. The primary cardiovascular outcomes will include: cardiovascular death; nonfatal myocardial infarction (i.e. unstable angina); and non-fatal stroke based on the FDA definition [32]. Secondary cardiovascular outcomes will include: transient ischemic attack; congestive heart failure; palpitations; arrhythmias; and thromboembolism (deep vein thrombosis and pulmonary embolism).

Other outcomes will include adverse events such as nausea, headache, dry mouth, skin rash and pruritus.

## Effectiveness analysis


For peer review only - http://bmjopen.bmj.com/site/about/guidelines.xhtml

The primary effectiveness outcome will be sustained smoking cessation, defined as abstinence for a minimum of six months as determined by biochemically validated continuous or prolonged abstinence at the longest reported time point in intention to treat analyses, as this is the strictest definition of abstinence. As we are using data from previous Cochrane reviews of smoking cessation medicines [33-36], we have also chosen this outcome to be consistent with the definitions used in those reviews. Where these data are not available, we will accept point prevalence abstinence and self-report quit data. We will treat participants who drop out or are lost to follow-up as continuing smokers. We will extract information on abstinence at each time point for which it is reported to allow a survival model to be estimated following the approach used by Chen*, et al.* [37], Madan*, et al.* [38]. Data on reductions in smoking, rather than abstinence, will not be included. Secondary outcomes will include reduction in craving and reduction in withdrawal symptoms.

## Search strategy

We will search the following databases: MEDLINE; EMBASE; PsycINFO; Web of Science; Clinicaltrials.gov; and the Cochrane Databases including the Cochrane Database of Systematic Reviews (CDSR), the Database of Abstracts and Reviews of Effectiveness (DARE) (updated until March 2015), the Cochrane Central Register of Controlled Trials (CENTRAL), the NHS Economic Evaluation Database (NHS EED) and the Health Technology Assessment Database (HTA). Searches will be conducted with the help of an information specialist and will not include any language restrictions. Non-English language articles will be reviewed by native speakers prior to obtaining a full translation. We will also manually search the reference lists of relevant research articles and previous reviews and communicate with authors in an attempt to identify unpublished information; in a previous study we had a 75% positive response rate from corresponding authors for studies published after 2006 [17]. We will also review the literature to identify disutilities and costs associated with neuropsychiatric and cardiovascular treatment-related adverse events. Acknowledging that we may not find many studies in a smoking cessation population, we will also search for studies reporting disutilities and costs for the same events in other populations.

To identify studies for the safety network meta-analyses we will build on the basic search strategy included in the cardiovascular network meta-analysis by Mills*, et al.* [39]. Online supplementary appendix 2 presents our full electronic search strategy for observational studies of varenicline in Medline. To identify studies for the effectiveness network meta-analyses the search strategies from four Cochrane reviews [33-36] (or updated versions where available) will be modified to identify more recent trials for inclusion in the current study in addition to their previously identified trials. Searches for observational studies will not be date-limited. We will adapt the Sheffield economic model [30] to incorporate disutilities associated with neuropsychiatric and cardiovascular treatment-related adverse events, and update model inputs. The searches used for the network-meta-analyses will be re-run with a cost-effectiveness filter to identify studies reporting information on utilities, disutilities, resource use, and costs, which will be used to inform the economic evaluation.



For peer review only - http://bmjopen.bmj.com/site/about/guidelines.xhtml

## Study selection and data extraction

Search results will be uploaded to Covidence [40], which we will use to screen abstracts and full text and to resolve disagreements. Reviewers (MND – all papers, KHT and DC – papers equally shared) will independently screen abstracts to determine whether full text reports should be obtained. The same reviewers will independently identify eligible full text reports for inclusion. Discrepancies will be resolved by reaching consensus amongst reviewers. We will include a PRISMA diagram [41] to set out the results of the searches and to indicate the number of included and excluded trials. The reasons for excluding studies following full text screening will be documented.

Data for studies included following the updated search will be extracted by one reviewer (MND) and checked by co-reviewers (KHT and DC). Information will be collected on: study design (duration of treatment, description of allocation concealment and blinding); study participants (inclusion and exclusion criteria, country, region, population studied); baseline characteristics (e.g., ethnicity, sex, smoking history); intervention and comparison groups (including the smoking cessation intervention, whether or not there was co-treatment, dosage and formulation); our predefined primary and secondary outcomes of interest including measures of effectiveness and safety outcomes; losses to follow up; and study sponsor. All of the extracted information will be summarised in tables. In the event of missing data, we will contact authors by email to ask for original data. Authors of all newly identified studies will be contacted to verify the accuracy of the extracted data.

## Risk of bias assessment

For RCTs the Cochrane tool for assessing the risk of bias [42] will be used to determine whether there is high, low, or unclear risk of bias in the following domains: random sequence generation; allocation concealment; blinding of participants and personnel; blinding of outcome assessment; incomplete outcome data; selective outcome reporting; and other sources of bias. For observational studies we will consider using the risk of bias in non-randomized studies of interventions (ROBINS-I) tool [43] to determine whether there is low, moderate, serious, or critical risk of bias or no information in the following domains: confounding; participant selection; intervention classification; intervention deviations; missing data; outcome measurement; and selection of the reported result. Reviewers (MND – all papers, KHT and DC – papers equally shared) will independently assess the risk of bias in each of the trials. Discrepancies will be resolved by referring to the original publication and reaching consensus amongst reviewers. Study authors will be contacted to obtain study protocols and additional information that may not have been published to aid with assessment of the risk of bias.

## Data synthesis

Effectiveness outcomes will be summarised as risk ratios. Because safety outcomes are rare, we will follow recommendations of Bradburn*, et al.* [44] and use Peto odds ratios to compare treatment groups although we will also report risk differences. All results will be reported including 95% confidence intervals. The pairwise and network



For peer review only - http://bmjopen.bmj.com/site/about/guidelines.xhtml

meta-analyses will be conducted in a Bayesian framework using OpenBUGS software, using code developed by Dias, *et al.* [45]. We will report results of both fixed- and random-effects meta-analyses; between-study heterogeneity will be quantified using the between-study standard deviation (τ). For each separate outcome, we will also construct a network meta-analysis (NMA) that will be compared to the pair-wise results. For the safety outcomes, separate analyses will be conducted for RCTs and observational studies. NMA is an extension of standard meta-analysis that allows evidence synthesis to be performed when there are multiple competing interventions available by simultaneously combining evidence from *all* trials reporting that outcome, so long as treatments form a connected network [46]. Figure 1 shows an example of a connected network of 7 smoking cessation treatments adapted from a Cochrane overview of reviews of smoking cessation at 6 months [14]. For the NMA combination therapies will be considered as separate interventions but we will explore models for the effects of the component therapies using a main effects model and a 2-way interaction model (allowing pairs of therapies to have either a bigger or smaller effect than would be expected from the sum of their effects alone). For the random effects NMA we will assume homogeneous between-study variability across studies. We will assess the goodness of fit of each model to the data by calculating the posterior mean residual deviance. This is defined as the difference between the deviance for the fitted model and the saturated model, where the deviance measures the fit of the model using the likelihood function. The Deviance Information Criterion (DIC), which is equal to the sum of the posterior mean of the residual deviance and the effective number of parameters pD, will be used as a basis for model comparison [47]. The DIC penalises the posterior mean residual deviance (a measure of model fit) by the effective number of parameters in the model (as measure of complexity) and can therefore be viewed as a trade-off between the fit and complexity of the model.

Figure 1. Network diagram for effectiveness of smoking cessation treatments. Adapted from [14].

Insert Figure 1 Here

For the pairwise analyses only, and where the number of studies allow, we will produce funnel plots.

**Assessment of the consistency assumption**

Validity of a NMA depends on the assumption that there is no effect modification of the pairwise intervention effects or, that the prevalence of effect modifiers is similar in the different studies. This key assumption has been referred to variously as transitivity [48], similarity [49] and consistency [50]. We will compile a table of important trial and patient characteristics and visually inspect the 'similarity' of factors we consider likely to modify treatment effect.

We will visually inspect the network diagram to identify the extent of potential inconsistency (the number of loops) and use model fit and selection statistics to informally assess whether it is evident. If inconsistency is suspected we will explore it formally using a "node-splitting" approach [50].



For peer review only - http://bmjopen.bmj.com/site/about/guidelines.xhtml

### Health economic modelling

A previously built model [30] will be used to evaluate cost-effectiveness using probabilistic methods and reporting fully incremental analyses that meet the NICE reference case [51]. This model has nine health states including those most commonly related to smoking. Where data allow the model will be adapted to allow more than one attempt at quitting and the addition of serious adverse neuropsychiatric and cardiovascular events.

Subgroup analyses will be presented as appropriate. Sensitivity analyses will be performed including and excluding smokeless tobacco users. We will use the Grading of Recommendations Assessment, Development and Evaluation (GRADE) approach to assess the quality of evidence [52].Evidence for both effectiveness and safety outcomes will be judged for the domains of risk of bias, consistency, precision, reporting bias and directness. Evidence will be ranked as being of high, moderate, low, or very low quality.

# CONCLUSION

The proposed systematic review, network meta-analysis and cost-effectiveness analysis will address important questions about the relative costs or risks and benefits of different smoking cessation pharmacotherapies. It is expected that the study findings will be incorporated into future updates of the new NICE Public Health guidance on Smoking Cessation Interventions and Services, a partial update of the NICE Public Health guidance on Smoking Cessation Services [4], expected to be published in November 2017. Critically, we believe that the study findings will offer patients, prescribers and regulators updated and comprehensive information on the safety and effectiveness of these smoking cessation medicines that will allow them to make informed decisions when evaluating or selecting a pharmacological treatment to assist with a quit attempt.

# ETHICS AND DISSEMINATION

Ethics approval is not required for this evidence synthesis study as it involves analysis of secondary data from randomised controlled trials and observational studies. We anticipate dissemination to the following groups, for whom the results of this research will be of interest: the general public; clinicians and healthcare practitioners; academics; policy makers; and industry. Findings from the study will be disseminated through conventional academic routes such as peer reviewed publications and presentations and regional, national and international conferences.

***Authors' contributions*** KHT, DC and NJW conceived the idea, planned and designed the study protocol. DC designed the network diagram. KHT, DC and NJW planned the data extraction and statistical analyses. NJW and MS planned the economic evaluation. MRM and DG provided critical insight. KHT and MND wrote the first draft of the protocol and all authors approved and contributed to the final manuscript. KHT is the guarantor of the review.



For peer review only - http://bmjopen.bmj.com/site/about/guidelines.xhtml

**Funding statement** This project presents independent research which was funded by the National Institute for Health Research Health Technology Assessment Programme (project number NIHR HTA 15/58/18). The views and opinions expressed therein are those of the authors and do not necessarily reflect those of the Health Technology Assessment Programme, NIHR, NHS or the Department of Health.

**Competing interests** MRM reports grants from Pfizer, grants from Rusan, and non-financial support from GlaxoSmithKline, outside the submitted work. DG had a specified relationship with the MHRA in the past (was a member of the MHRA's Pharmacovigilance Expert Advisory Group and received travel expenses and a small fee for meeting attendance and preparation for meetings).

# References

1. Peto R, Darby S, Deo H, Silcocks P, Whitley E, Doll R. Smoking, smoking cessation, and lung cancer in the UK since 1950: combination of national statistics with two case-control studies. BMJ 2000;**321**(7257):323-9
2. Jha P, Peto R. Global Effects of Smoking, of Quitting, and of Taxing Tobacco. N Engl J Med 2014;**370**(1):60-68 doi: doi:10.1056/NEJMra1308383[published Online First: Epub Date]|.
3. Health and Social Care Information Centre. Statistics on Smoking: England, 2012, 2012.
4. National Institute for Health and Clinical Excellence. NICE public health guidance 10. Smoking cessation services in primary care, pharmacies, local authorities and workplaces, particularly for manual working groups, pregnant women and hard to reach communities. London: NICE, 2008.
5. Medicines & Healthcare products Regulatory Agency. Public Assessment Report. e-Voke 10mg and 15mg Electronic Inhaler (PL 42601/0003-4). Secondary Public Assessment Report. e-Voke 10mg and 15mg Electronic Inhaler (PL 42601/0003-4) 2015. http://www.mhra.gov.uk/home/groups/par/documents/websiteresources/con616843.pdf.
6. Medicines and Healthcare products Regulatory Agency. Varenicline:adverse psychiatric reactions, including depression. Drug Safety Update 2008;**2**(4):2-3
7. US Food and Drug Administration. Information for Healthcare Professionals: Varenicline (marketed as Chantix) and Bupropion (marketed as Zyban, Wellbutrin and generics). FDA Drug Safety Newsletter 2009; 2(1). http://www.fda.gov/Drugs/DrugSafety/PostmarketDrugSafetyInformationforPatientsandProviders/DrugSafetyInformationforHeathcareProfessionals/ucm169986.htm.
8. Harrison-Woolrych M. Mental health effects of varenicline. BMJ : British Medical Journal 2015;**350**
9. Anthenelli RM, Benowitz NL, West R, et al. Neuropsychiatric safety and efficacy of varenicline, bupropion, and nicotine patch in smokers with and without psychiatric disorders (EAGLES): a double-blind, randomised, placebo-controlled clinical trial. The Lancet 2016;**387**(10037):2507-20 doi: 10.1016/S0140-6736(16)30272-0[published Online First: Epub Date]|.
10. European Medicines Agency (EMA). Champix Procedural steps taken and scientific information after the authorization. . Secondary Champix Procedural steps taken and scientific information after the authorization. 2016. http://www.ema.europa.eu/docs/en_GB/document_library/EPAR_-



For peer review only - http://bmjopen.bmj.com/site/about/guidelines.xhtml

_Procedural_steps_taken_and_scientific_information_after_authorisation/human/000699/WC500025256.pdf
11. Thomas KH, Martin RM, Davies N, Metcalfe C, Windmeijer F, Gunnell D. Smoking cessation treatment and the risk of depression, suicide and self-harm in the Clinical Practice Research Datalink: prospective cohort study BMJ 2013;**347**:f5704
12. Kotz D, Viechtbauer W, Simpson C, van Schayck OCP, West R, Sheikh A. Cardiovascular and neuropsychiatric risks of varenicline: a retrospective cohort study. The Lancet Respiratory Medicine 2015;**3**(10):761-68 doi: http://dx.doi.org/10.1016/S2213-2600(15)00320-3[published Online First: Epub Date]|.
13. Action on Smoking and Health. Use of electronic cigarettes (vapourisers) among adults in Great Britain, 2016.
14. Cahill K, Stevens S, Perera R, Lancaster T. Pharmacological interventions for smoking cessation: an overview and network meta-analysis. Cochrane Database Syst Rev 2013;**5** doi: 10.1002/14651858.CD009329.pub2[published Online First: Epub Date]|.
15. Prescribing and Primary Care team Health and Social Care Information Centre. Prescription Cost Analysis, England, 2013. 2014. http://www.hscic.gov.uk/catalogue/PUB13887/pres-cost-anal-eng-2013-rep.pdf.
16. Tonstad S, Davies S, Flammer M, Russ C, Hughes J. Psychiatric Adverse Events in Randomized, Double-Blind, Placebo-Controlled Clinical Trials of Varenicline. Drug-Safety 2010;**33**(4):289-301 doi: 10.2165/11319180-000000000-00000[published Online First: Epub Date]|.
17. Thomas KH, Martin RM, Knipe DW, Higgins JP, Gunnell D. Risk of neuropsychiatric adverse events associated with varenicline: systematic review and meta-analysis. BMJ 2015;**350**:h1109 doi: 10.1136/bmj.h1109[published Online First: Epub Date]|.
18. Gibbons RD, Mann JJ. Varenicline, Smoking Cessation, and Neuropsychiatric Adverse Events. The American journal of psychiatry 2013 doi: 10.1176/appi.ajp.2013.12121599[published Online First: Epub Date]|.
19. Moore TJ, Furberg CD, Glenmullen J, Maltsberger JT, Singh S. Suicidal Behavior and Depression in Smoking Cessation Treatments. Plos One 2011;**6**(11):e27016 doi: 10.1371/journal.pone.0027016[published Online First: Epub Date]|.
20. Harrison-Woolrych M, Ashton J. Psychiatric Adverse Events Associated with Varenicline: An Intensive Postmarketing Prospective Cohort Study in New Zealand. Drug Saf 2011;**34**(9):763-72
21. Kasliwal R, Wilton LV, Shakir SAW. Safety and Drug Utilization Profile of Varenicline as Used in General Practice in England: Interim Results from a Prescription-Event Monitoring Study. Drug Saf 2009;**32**(6):499-507 doi: 10.2165/00002018-200932060-00006[published Online First: Epub Date]|.
22. Gunnell D, Irvine D, Wise L, Davies C, Martin RM. Varenicline and suicidal behaviour: a cohort study based on data from the General Practice Research Database. Brit Med J 2009;**339** doi: Artn B3805 Doi 10.1136/Bmj.B3805[published Online First: Epub Date]|.
23. Meyer TE, Taylor LG, Xie S, et al. Neuropsychiatric events in varenicline and nicotine replacement patch users in the Military Health System. Addiction 2013;**108**(1):203-10 doi: 10.1111/j.1360-0443.2012.04024.x[published Online First: Epub Date]|.
24. Campbell AR, Anderson KD. Mental health stability in veterans with posttraumatic stress disorder receiving varenicline. American journal of health-system pharmacy : AJHP : official journal of the American Society of Health-System Pharmacists 2010;**67**(21):1832-7 doi: 10.2146/ajhp100196[published Online First: Epub Date]|.



For peer review only - http://bmjopen.bmj.com/site/about/guidelines.xhtml

25. Thomas KH, Martin RM, Potokar J, Pirmohamed M, Gunnell D. Reporting of drug induced depression and fatal and non-fatal suicidal behaviour in the UK from 1998 to 2011. BMC Pharmacology and Toxicology 2014;**15**(1):1-11 doi: 10.1186/2050-6511-15-54[published Online First: Epub Date]|.
26. Davis S, King B, Raine JM. Spontaneous reporting- UK. In: Mann RD, Andrews EB, eds. Pharmacovigilance: Second Edition Chichester, West Sussex: John Wiley & Sons, 2007:199-215.
27. Martin RM, May M, Gunnell D. Did intense adverse media publicity impact on prescribing of paroxetine and the notification of suspected adverse drug reactions? Analysis of routine databases, 2001-2004. Br J Clin Pharmacol 2006;**61**:224-28
28. Walker AM. Confounding by indication. Epidemiology (Cambridge, Mass.) 1996;**7**(4):335-6
29. US Department of Health and Human Services. The Health Consequences of Smoking-50 Years of Progress: A Report of the Surgeon General. Atlanta (GA): US Department of Health and Human Services, Centers for Disease Control and Prevention, National Center for Chronic Disease Prevention, Health Promotion Office on Smoking Health, 2014.
30. Leaviss J, Sullivan W, Ren S, et al. What is the clinical effectiveness and cost-effectiveness of cytisine compared with varenicline for smoking cessation? A systematic review and economic evaluation. Health technology assessment (Winchester, England) 2014;**18**(33):1-120 doi: 10.3310/hta18330[published Online First: Epub Date]|.
31. Nakamura M, Oshima A, Fujimoto Y, Maruyama N, Ishibashi T, Reeves KR. Efficacy and tolerability of varenicline, an alpha(4)beta(2) nicotinic acetylcholine receptor partial agonist, in a 12-week, randomized, placebo-controlled, dose-response study with 40-week follow-up for smoking cessation in Japanese smokers. Clinical Therapeutics 2007;**29**(6):1040-56 doi: 10.1016/j.clinthera.2007.06.012[published Online First: Epub Date]|.
32. Hertzberg MA, Moore SD, Feldman ME, Beckham JC. A preliminary study of bupropion sustained-release for smoking cessation in patients with chronic posttraumatic stress disorder. Journal of clinical psychopharmacology 2001;**21**(1):94-8
33. Cahill K, Lindson-Hawley N, Thomas KH, Fanshawe TR, Lancaster T. Nicotine receptor partial agonists for smoking cessation. Cochrane Database of Systematic Reviews 2016(5) doi: 10.1002/14651858.CD006103.pub7[published Online First: Epub Date]|.
34. Hughes J, R., Stead LF, Hartmann-Boyce J, Cahill K, Lancaster T. Antidepressants for smoking cessation. Cochrane Database of Systematic Reviews 2014(1) doi: 10.1002/14651858.CD000031.pub4[published Online First: Epub Date]|.
35. Stead LF, Perera R, Bullen C, et al. Nicotine replacement therapy for smoking cessation. Cochrane database of systematic reviews (Online) 2012;**11**
36. Hartmann-Boyce J, McRobbie H, Bullen C, Begh R, Stead LF, Hajek P. Electronic cigarettes for smoking cessation. Cochrane Database of Systematic Reviews 2016(9) doi: 10.1002/14651858.CD010216.pub3[published Online First: Epub Date]|.
37. Chen YF, Madan J, Welton N, et al. Effectiveness and cost-effectiveness of computer and other electronic aids for smoking cessation: a systematic review and network meta-analysis. Health technology assessment (Winchester, England) 2012;**16**(38):1-205, iii-v doi: 10.3310/hta16380[published Online First: Epub Date]|.
38. Madan J, Chen Y-F, Aveyard P, et al. Synthesis of evidence on heterogeneous interventions with multiple outcomes recorded over multiple follow-up times reported inconsistently: a smoking cessation case-study. Journal of the Royal Statistical



For peer review only - http://bmjopen.bmj.com/site/about/guidelines.xhtml

Society: Series A (Statistics in Society) 2014;**177**(1):295-314 doi: 10.1111/rssa.12018[published Online First: Epub Date]|.
39. Mills EJ, Thorlund K, Eapen S, Wu P, Prochaska JJ. Cardiovascular events associated with smoking cessation pharmacotherapies: a network meta-analysis. Circulation 2014;**129**(1):28-41 doi: 10.1161/circulationaha.113.003961[published Online First: Epub Date]|.
40. Covidence systematic review software. Melbourne, Australia: Veritas Health Innovation. Available at http://www.covidence.org/
41. Moher D, Liberati A, Tetzlaff J, Altman DG. Preferred reporting items for systematic reviews and meta-analyses: the PRISMA statement. BMJ 2009;**339** doi: 10.1136/bmj.b2535[published Online First: Epub Date]|.
42. Higgins JPT, Altman DG, Gøtzsche PC, et al. The Cochrane Collaboration's tool for assessing risk of bias in randomised trials. BMJ 2011;**343**
43. Sterne JAC, Hernán MA, Reeves BC, et al. ROBINS-I: a tool for assessing risk of bias in non-randomised studies of interventions. BMJ 2016;**355**
44. Bradburn MJ, Deeks JJ, Berlin JA, Russell Localio A. Much ado about nothing: a comparison of the performance of meta-analytical methods with rare events. Statistics in medicine 2007;**26**(1):53-77 doi: 10.1002/sim.2528[published Online First: Epub Date]|.
45. Dias S, Sutton AJ, Ades AE, Welton NJ. Evidence Synthesis for Decision Making 2: A Generalized Linear Modeling Framework for Pairwise and Network Meta-analysis of Randomized Controlled Trials. Medical Decision Making 2013;**33**(5):607-17 doi: 10.1177/0272989x12458724[published Online First: Epub Date]|.
46. Caldwell DM, Ades AE, Higgins JPT. Simultaneous comparison of multiple treatments: combining direct and indirect evidence. BMJ 2005;**331**(7521):897
47. Spiegelhalter DJ, Best NG, Carlin BP, Van Der Linde A. Bayesian measures of model complexity and fit. Journal of the Royal Statistical Society: Series B (Statistical Methodology) 2002;**64**(4):583-639 doi: 10.1111/1467-9868.00353[published Online First: Epub Date]|.
48. Salanti G, Higgins JP, Ades A, Ioannidis JP. Evaluation of networks of randomized trials. Statistical Methods in Medical Research 2008;**17**(3):279-301 doi: 10.1177/0962280207080643[published Online First: Epub Date]|.
49. Song F, Altman DG, Glenny AM, Deeks JJ. Validity of indirect comparison for estimating efficacy of competing interventions: empirical evidence from published meta-analyses. Bmj 2003;**326**(7387):472 doi: 10.1136/bmj.326.7387.472[published Online First: Epub Date]|.
50. Dias S, Welton NJ, Caldwell DM, Ades AE. Checking consistency in mixed treatment comparison meta-analysis. Statistics in medicine 2010;**29**(7-8):932-44 doi: 10.1002/sim.3767[published Online First: Epub Date]|.
51. National Institute for Health and Clinical Excellence. Guide to the Methods of Technology Appraisal 2013. London: NICE, 2013.
52. Guyatt GH, Oxman AD, Vist GE, et al. GRADE: an emerging consensus on rating quality of evidence and strength of recommendations. BMJ 2008;**336**(7650):924-26 doi: 10.1136/bmj.39489.470347.AD[published Online First: Epub Date]|.



For peer review only - http://bmjopen.bmj.com/site/about/guidelines.xhtml

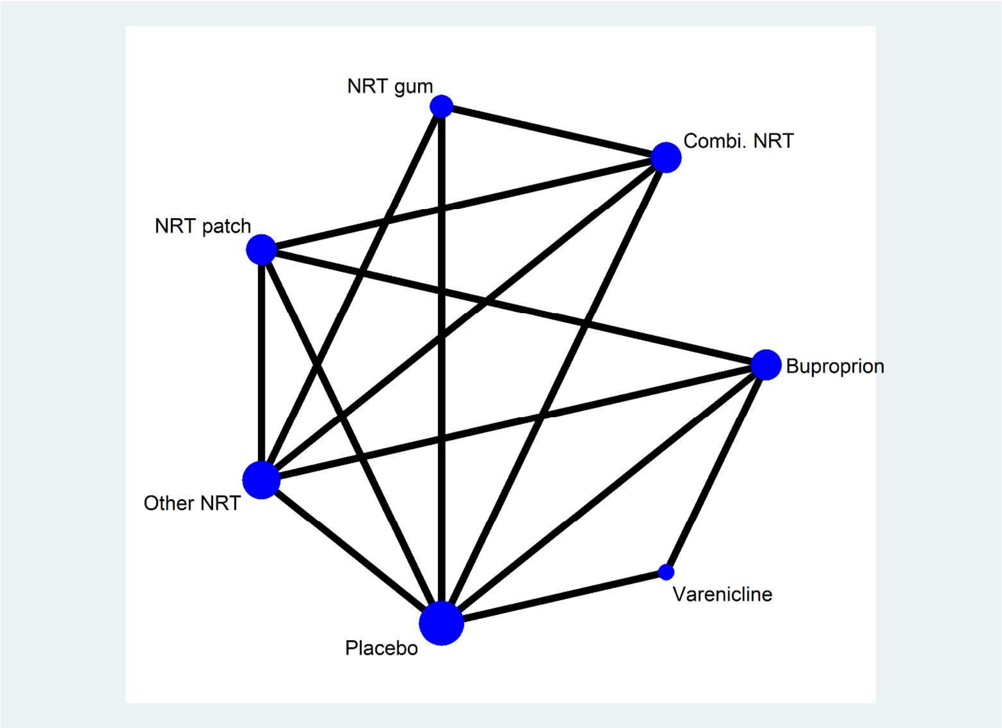


Note. The circles represent treatment “nodes” and the solid lines denote the presence of direct trial evidence comparing two treatments. Line thickness is proportional to the number of trials; the thicker the line, the more trials have compared treatments.

338x246mm (300 x 300 DPI)

# PRISMA-P Checklist

This checklist has been adapted for use with protocol submissions to *Systematic Reviews* from Table 3 in Moher D et al: Preferred reporting items for systematic review and meta-analysis protocols (PRISMA-P) 2015 statement. *Systematic Reviews* 2015 **4**:1

| Section/topic | # | Checklist item | Information reported | | Page number(s) |
|---|---|---|---|---|---|
| | | | Yes | No | |
| **ADMINISTRATIVE INFORMATION** | | | | | |
| **Title** | | | | | |
| Identification | 1a | Identify the report as a protocol of a systematic review | ☒ | ☐ | 1 |
| Update | 1b | If the protocol is for an update of a previous systematic review, identify as such | ☐ | ☒ | |
| **Registration** | 2 | If registered, provide the name of the registry (e.g., PROSPERO) and registration number in the Abstract | ☒ | ☐ | 2 |
| **Authors** | | | | | |
| Contact | 3a | Provide name, institutional affiliation, and e-mail address of all protocol authors; provide physical mailing address of corresponding author | ☒ | ☐ | 1 |
| Contributions | 3b | Describe contributions of protocol authors and identify the guarantor of the review | ☒ | ☐ | 10 |
| **Amendments** | 4 | If the protocol represents an amendment of a previously completed or published protocol, identify as such and list changes; otherwise, state plan for documenting important protocol amendments | ☐ | ☒ | |
| **Support** | | | | | |
| Sources | 5a | Indicate sources of financial or other support for the review | ☒ | ☐ | 11 |
| Sponsor | 5b | Provide name for the review funder and/or sponsor | ☒ | ☐ | 11 |
| Role of sponsor/funder | 5c | Describe roles of funder(s), sponsor(s), and/or institution(s), if any, in developing the protocol | ☒ | ☐ | 11 |
| **INTRODUCTION** | | | | | |
| **Rationale** | 6 | Describe the rationale for the review in the context of what is already known | ☒ | ☐ | 3-5 |
| **Objectives** | 7 | Provide an explicit statement of the question(s) the review will address with reference to participants, interventions, comparators, and outcomes (PICO) | ☒ | ☐ | 5-7 |

For peer review only

| Section/topic | # | Checklist item | Information reported | | Page number(s) |
|---|---|---|---|---|---|
| | | | Yes | No | |
| | | | | | |
| **METHODS** | | | | | |
| **Eligibility criteria** | 8 | Specify the study characteristics (e.g., PICO, study design, setting, time frame) and report characteristics (e.g., years considered, language, publication status) to be used as criteria for eligibility for the review | ☒ | ☐ | 5-7 |
| **Information sources** | 9 | Describe all intended information sources (e.g., electronic databases, contact with study authors, trial registers, or other grey literature sources) with planned dates of coverage | ☒ | ☐ | 7 |
| **Search strategy** | 10 | Present draft of search strategy to be used for at least one electronic database, including planned limits, such that it could be repeated | ☒ | ☐ | Supplementary File Appendix 2 |
| ***STUDY RECORDS*** | | | | | |
| Data management | 11a | Describe the mechanism(s) that will be used to manage records and data throughout the review | ☒ | ☐ | 8 |
| Selection process | 11b | State the process that will be used for selecting studies (e.g., two independent reviewers) through each phase of the review (i.e., screening, eligibility, and inclusion in meta-analysis) | ☒ | ☐ | 8 |
| Data collection process | 11c | Describe planned method of extracting data from reports (e.g., piloting forms, done independently, in duplicate), any processes for obtaining and confirming data from investigators | ☒ | ☐ | 8 |
| **Data items** | 12 | List and define all variables for which data will be sought (e.g., PICO items, funding sources), any pre-planned data assumptions and simplifications | ☒ | ☐ | 8 |
| **Outcomes and prioritization** | 13 | List and define all outcomes for which data will be sought, including prioritization of main and additional outcomes, with rationale | ☒ | ☐ | 6-7 |
| **Risk of bias in individual studies** | 14 | Describe anticipated methods for assessing risk of bias of individual studies, including whether this will be done at the outcome or study level, or both; state how this information will be used in data synthesis | ☒ | ☐ | 8 |
| ***DATA*** | | | | | |
| **Synthesis** | 15a | Describe criteria under which study data will be quantitatively synthesized | ☒ | ☐ | 8-9 |
| | 15b | If data are appropriate for quantitative synthesis, describe planned summary measures, methods of handling data, and methods of combining data from studies, including any planned exploration of consistency (e.g., $I^2$, Kendall's tau) | ☒ | ☐ | 8-10 |
| | 15c | Describe any proposed additional analyses (e.g., sensitivity or subgroup analyses, meta-regression) | ☒ | ☐ | 10 |
| | 15d | If quantitative synthesis is not appropriate, describe the type of summary planned | ☐ | ☒ | |

For peer review only

| Section/topic | # | Checklist item | Information reported | | Page number(s) |
|---|---|---|---|---|---|
| | | | Yes | No | |
| **Meta-bias(es)** | 16 | Specify any planned assessment of meta-bias(es) (e.g., publication bias across studies, selective reporting within studies) | ☒ | ☐ | 9 |
| **Confidence in cumulative evidence** | 17 | Describe how the strength of the body of evidence will be assessed (e.g., GRADE) | ☒ | ☐ | 10 |

For peer review only

Appendix 1. Summary of pharmacologic interventions by dose and formulation.

| Treatment and formulation | Lower dose | Standard dose | Higher dose |
|---|---|---|---|
| *Bupropion* | | | |
| Oral extended release tablets | <150 mg bd | 150 mg bd | >150 mg bd |
| *Varenicline* | | | |
| Tablets | <1 mg bd | 1 mg bd | >1 mg bd |
| *Nicotine replacement therapy* | | | |
| Patch (16 hrs) | <15 mg (5 mg/16 hours or 10 mg/16 hours) | 15 mg (15 mg/16 hours) | >15 mg (25 mg/16 hours) |
| Patch (24 hrs) | < 14 mg (7 mg/24 hours) | 14 mg (14 mg/24 hours) | >14 mg (21 mg/24 hours) |
| Gum | | 2 mg/piece (15 pieces daily) | 4 mg/piece (15 pieces daily) |
| Nasal spray | | 0.5 mg/metered spray (up to 2 sprays/hour, 64 daily) | |
| Mouth spray | | 1 mg/metered spray (up to 4 sprays/hour, 64 daily) | |
| Lozenge | 1 mg or 1.5 mg (1 lozenge/1-2 hours, 15 daily) | 2 mg (1 lozenge/1-2 hours, 15 daily) | 4 mg (1 lozenge/1-2 hours, 15 daily) |
| Sublingual tablet | | 2 mg per tablet (up to 1 tablet/hour, 40 daily) | 2 mg per tablet (up to 2 tablets/hour, 40 daily) |
| Inhalator | | 10 mg/cartridge (12 cartridges daily) | 15 mg/cartridge (6 cartridges daily) |
| *Electronic Cigarette* | | | |
| Electronic Inhaler | | 10mg/cartridge (5 cartridges daily) | 15mg/cartridge (5 cartridges daily) |

*Note*. Maximum doses in parentheses; mg = milligram; bd = twice daily. For nicotine replacement therapy the higher dose is generally used in smokers who smoke >20 cigarettes per day.

For peer review only



For peer review only - http://bmjopen.bmj.com/site/about/guidelines.xhtml

Appendix 2. Full electronic search strategy for observational studies of varenicline in Medline.

1 Smoking/

2 Tobacco/

3 Nicotine/

4 Tobacco Products/

5 Smoking/dt [Drug Therapy]

6 Smoking Cessation/

7 "Tobacco Use Cessation"/

8 "Tobacco Use Disorder"/

9 Smoking/pc [Prevention & Control]

10 (smoking or smoker*).ti,ab,kf.

11 (tobacco* or cigar* or cigarette* or nicotine).ti,ab,kf.

12 ((smoking or tobacco) adj5 (cessation or ceas* or quit* or stop* or giv* or prevent* or abstain* or abstin* or control*)).ti,ab,kf.

13 Varenicline/

14 Nicotinic Agonists/

15 varenicline.ti,ab,kf.

16 (champix or tabex or chantix).ti,ab,kf.

17 (nicotinic adj3 agonist*).ti,ab,kf.

18 (benzazepine* adj2 derivative*).ti,ab,kf.

19 nicotinic receptor partial agonist*.ti,ab,kf.

20 Epidemiologic Methods/ (30872)

21 epidemiologic studies/

22 case-control studies/ or retrospective studies/

23 cohort studies/ or follow-up studies/ or longitudinal studies/ or "national longitudinal study of adolescent health"/ or prospective studies/ or retrospective studies/

24 case-control studies/

25 Cross-Sectional Studies/

26 case control.ti,ab,kf.

27 (cohort adj (study or studies)).ti,ab,kf.



For peer review only - http://bmjopen.bmj.com/site/about/guidelines.xhtml

28 cohort analy*.ti,ab,kf.

29 (follow-up adj (study or studies)).ti,ab,kf.

30 (observational adj (study or studies)).ti,ab,kf.

31 (longitudinal or retrospective or prospective or cross sectional).ti,ab,kf.

32 (epidemiologic* adj (study or studies)).ti,ab,kf.

33 1 or 2 or 3 or 4 or 5 or 6 or 7 or 8 or 9 or 10 or 11 or 12

34 13 or 14 or 15 or 16 or 17 or 18 or 19

35 20 or 21 or 22 or 23 or 24 or 25 or 26 or 27 or 28 or 29 or 30 or 31 or 32

36 33 and 34 and 35

For peer review only



For peer review only - http://bmjopen.bmj.com/site/about/guidelines.xhtml
